# A Composite Bioinformatic Analysis to Explore Endoplasmic Reticulum Stress-Related Prognostic Marker and Potential Pathogenic Mechanisms in Glioma by Integrating Multiomics Data

**DOI:** 10.1155/2022/9886044

**Published:** 2022-10-04

**Authors:** Xin Fan, Xiyi Nie, Junwen Huang, Lingling Zhang, Xifu Wang, Min Lu

**Affiliations:** ^1^Department of Emergency, Shangrao Hospital Affiliated to Nanchang University, Shangrao People's Hospital, Shangrao 334000, China; ^2^Department of Otolaryngology-Head and Neck Surgery, The First Affiliated Hospital of Nanchang University, Nanchang 330000, China; ^3^Department of Neurosurgery, Yichun Hospital Affiliated to Nanchang University, Yichun People's Hospital, Yichun 334000, China; ^4^The First Clinical Medical College of Nanchang University, Nanchang 330000, China; ^5^School of Stomatology, Nanchang University, Nanchang 330000, China

## Abstract

In recent years, abnormal endoplasmic reticulum stress (ERS) response, as an important regulator of immunity, may play a vital role in the occurrence, development, and treatment of glioma. Weighted correlation network analysis (WGCNA) based on six glioma datasets was used to screen eight prognostic-related differentially expressed ERS-related genes (PR-DE-ERSGs) and to construct a prognostic model. BMP2 and HEY2 were identified as protective factors (HR < 1), and NUP107, DRAM1, F2R, PXDN, RNF19A, and SCG5 were identified as risk factors for glioma (HR > 1). QRT-PCR further supported significantly higher DRAM1 and lower SCG5 relative mRNA expression in gliomas. Our model has demonstrated excellent performance in predicting the prognosis of glioma patients from numerous datasets. In addition, the model shows good stability in multiple tests. Our model also shows broad clinical promise in predicting drug treatment effects. More immune cells/processes in the high-risk population with poor prognosis illustrate the importance of the tumor immunosuppressive environment in glioma. The potential role of the HEY2-based competitive endogenous RNA (ceRNA) regulatory network in glioma was validated and revealed the possible important role of glycolysis in glioma ERS. IDH1 and TP53 mutations with better prognosis were strongly associated with the risk score and PR-DE-ERSGs expression in the model. mDNAsi was also closely related to the risk score and clinical characteristics.

## 1. Introduction

Gliomas are the most common primary intracranial tumor, accounting for 81% of malignant brain tumor [1]. It is a very invasive brain tumor, which causes significant mortality and morbidity [2, 3]. With the development of molecular biology techniques, much evidence shows that gliomas' genetic and epigenetic features have changed significantly, facilitating many new diagnostic and therapeutic approaches, including targeted therapy and immunotherapy. Conventional therapies, including surgery, chemotherapy, and radiotherapy, have achieved limited improvements in the prognosis of glioma patients [4, 5]. Compared with other solid tumors, gliomas pose significant challenges to the development of novel tumor treatments due to biological factors such as the blood-brain barrier and unique tumor and immune microenvironments [6]. A remarkable level of genetic, epigenetic, and environmental heterogeneity exists within each glioma, providing multiple mechanisms of therapeutic resistance and forming this highly adaptable and resilient disease [7]. The current rapid development of in-silico technology provides new computational strategies for potential drug targets for many diseases [8–10]. Therefore, in order to improve the level of diagnosis and treatment of glioma and prognosis prediction, it is particularly necessary to identify effective biomarkers combined with clinical features.

The endoplasmic reticulum (ER) is the primary site for protein synthesis, processing, and trafficking, and many genetic and environmental impairments impede the cell's ability to fold correctly and post-translationally modify secreted and transmembrane proteins in the ER, resulting in the accumulation of misfolded proteins in the ER [11]. The above situation is called ER stress (ERS). Beyond a tolerable threshold, the accumulation of misfolded proteins triggers the unfolded protein response (UPR) to improve the folding ability of ER proteins during transcription and translation [12]. Robust ERS responses have been documented in most major types of human cancer, including breast, pancreatic, lung, skin, prostate, brain, and liquid malignancies [11]. Hostile microenvironmental conditions within tumor masses, such as nutrient deprivation, oxygen limitation, high metabolic demand, and oxidative stress, disturb the protein folding capacity of the ER, thereby provoking a cellular state of ERS [13]. Sustained activation of ERS sensors endows malignant cells with greater tumorigenic, metastatic, and drug resistant capacity [14].

Many studies have also reported the pathogenic role of ERS in the gliomas' initiation and progression and the potential therapeutic guiding value. ERS has been reported to be associated with self-renewal, differentiation, and drug resistance of glioma stem cells, and plays a vital role in the resistance of glioblastoma (GBM) to temozolomide toxicity (TMZ) [15–17]. General UPR activators or selective GRP78, ATF6, and PERK inducers have been detected to modulate cell proliferation and induce apoptosis in glioma cells [18]. Additionally, increased UPR activity strongly affects many intracellular metabolic pathways, which in turn shape the tumor microenvironment [18]. UPR favors increased 13C-glucose uptake and results in higher levels of lactate, alanine, and uridine diphosphate glucose, which are often associated with tumor aggressiveness [19]. Recent studies have shown that abnormal ERS response emerges as critical regulators of immune cell function in the tumor microenvironment, and it can subvert the protective function of innate immune cells in the tumor microenvironment to cripple the development of antitumor immunity [14]. Therefore, understanding the relationship between ERS and tumor microenvironment/immune cells and exploring the related mechanisms may become a new way to supplement and improve standard chemotherapy and immunotherapy in the clinic.

The competing endogenous RNA (ceRNA) hypothesis postulates that any RNA transcript with microRNA (miRNA)-response elements (MREs) can sequester miRNAs from other targets sharing the same MREs, thereby regulating their expression [20]. Therefore, cross-talk between RNAs, both coding and noncoding, through MREs forms a large-scale regulatory network across the transcriptome [21]. Mounting evidence has shown that various types of RNAs, including pseudogenes, long noncoding RNAs (lncRNAs), circular RNAs, and messenger RNAs, can function as ceRNAs in distinct physiological and pathophysiological states [22]. Recent studies on solid tumors and hematopoietic malignancies have shown that ceRNAs play significant roles in cancer pathogenesis by altering the expression of key tumorigenic or tumor-suppressive genes [23], including gastric cancer [24], lung adenocarcinoma [25], pancreatic cancer [26], and gallbladder [27]. While many studies investigate tumors, including gliomas, from the perspective of ERS, few studies have investigated gliomas from the perspective of ERS gene-related ceRNA. Therefore, exploring ERS-related ceRNA regulatory networks may contribute to the understanding of ERS-related biological roles in gliomas.

In this study, with the primary purpose of constructing a biomarker with high predictive performance and clinical value, we deeply analyzed ERS-related genes that are highly correlated with glioma progression and prognosis. To further elucidate the important role of ERS in glioma, a multi-omics analysis from the perspective of ERS-related immune microenvironment, cell stemness, and ceRNA regulatory network was also included in this study.

## 2. Material and Methods

### 2.1. Recruitment of Glioma Samples for Obtaining DE-ERSGs

**Figure 1:**
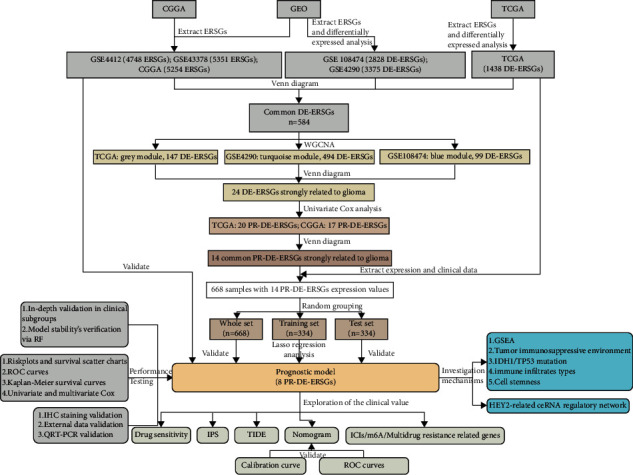
The schematic overview of our research process.

The schematic overview of our research process is shown in Figure 1. Our research enrolled the RNA sequencing data and clinical information of glioma samples from 3 public databases, including The Cancer Genome Atlas database (TCGA), Gene Expression Omnibus (GEO), and Chinese Glioma Genome Atlas (CGGA). The TCGA and CGGA databases contain 703 samples (698 glioma and 5 adjacent normal brain tissues) and 1018 glioma samples, respectively. The following 4 datasets from the GEO database were also included: GSE108474 (376 astrocytoma, 67 oligodendroglioma, and 28 normal brain tissues), GSE4290 (26 astrocytoma, 81 glioblastoma, 50 oligodendroglioma, and 23 normal brain tissues), GSE4412 (85 glioma), and GSE43378 (5 astrocytoma, 32 glioblastoma, and 13 anaplastic oligoastrocytoma).

In the meantime, we obtained 5909 endoplasmic reticulum stress-related genes (ERSGs) with a relevance score ≥1 from the gene card database (GeneCards), which contains genomic, transcriptomic, proteomic, genetic, clinical, and functional information integrated from 150 web sources [28]. Subsequently, we obtained the RNA sequencing level of ERSGs in those 6 datasets (TCGA: 5828, GSE108474: 5353, GSE4290: 5353, GSE4412: 4748, GSE43378: 5351, CGGA: 5254).

To obtain the differentially expressed endoplasmic reticulum stress-related genes (DE-ERSGs) between gliomas and normal tissues, we set filter conditions for datasets from different databases (TCGA: |log 2 fold change| (|log 2FC|) > 1 and false discovery rate (FDR) < 0.05, GEO: FDR < 0.05), respectively. We intersected DE-ERSGs from TCGA, GSE108474, and GSE4290 datasets with ERSGs from GSE4412, GSE43378, and CGGA datasets to obtain common genes. This process was shown via the R “Venn” package.

### 2.2. Screening for DE-ERSGs Strongly Related to Glioma

Weighted gene coexpression network analysis (WGCNA) was used to explore the modules of highly correlated genes among samples for relating modules to external sample traits [29]. We identified 5, 3, and 2 gene modules by WGCNA for the TCGA, GSE108474, and GSE4290 datasets, respectively. The values of best soft power *β* used in these three WGCNA processes are 11, 3, and 1, respectively. Among all modules, the turquoise module of GSE4290 has the strongest correlation with glioma. Therefore, we extracted DE-ERSGs strongly related to glioma from the modules with the strongest positive correlation with glioma from the TCGA, GSE108474, and GSE4290 datasets, respectively. The R “Venn” package was again used to obtain the common genes of the three gene modules.

### 2.3. Screening PR-DE-ERSGs for Prognostic Model's Construction Based on LASSO Regression

For subsequent survival analysis, we curated data from 6 datasets to extract glioma samples with full overall survival (OS) data. TCGA dataset's 668, GSE4412 dataset's 85, GSE43378 dataset's 50, and CGGA dataset's 983 glioma samples satisfy this requirement, as counted in Tables 1 and 2. Univariate Cox analyses identified differentially expressed endoplasmic reticulum stress-related genes associated with prognosis (PR-DE-ERSGs) based on TCGA and CGGA datasets data and filter threshold (*p* < 0.05), respectively. Common PR-DE-ERSGs from two datasets were extracted. Expression heatmap, forest plot reflecting univariate Cox regression results, and Kaplan–Meier survival curves based on TCGA data were all used to display the 14 common PR-DE-ERSGs.

The 668 TCGA samples were randomly equally divided into 2 subsets (training and test sets). Based on the RNA sequencing level of common PR-DE-ERSGs from the training set, the least absolute shrinkage and selection operator (LASSO) regression analysis was utilized to screen out highly related genes and minimize the risk of overfitting for screening signatures [30]. Eventually, 8 PR-DE-ERSGs and corresponding coefficients were obtained for constructing the prognostic model by calculating the optimal penalty parameter (*λ*). The risk score for each sample = sum of corresponding coefficients × matching gene's RNA sequencing level.

### 2.4. Validation of Differential Expression of 8 Genes by External Data

To maintain the stability of the model, we tried to validate the differential expression of eight genes in the model between glioma and normal brain tissues using data from an external database. GEPIA is a web tool server for cancer and normal gene expression profiling and interactive analyses [31]. Finally, we used this database to compare the eight gene expression differences between glioma (glioblastoma multiforme + lower grade glioma) and normal brain tissues.

### 2.5. Model's Performance and Stability Testing

After assigning a risk score for each sample, we got the median risk score. This median risk score directly split all samples into high-risk and low-risk groups. Risk curve and survival point plot were matched to show each sample's risk score and survival status. Kaplan–Meier survival curve and receiver operator characteristic (ROC) curve were used to test the performance of the model to discriminate and predict prognosis, respectively. Univariate and multivariate Cox regression analyses were used to determine the stability of the model as a prognostic factor before and after adjustment for confounders. To maintain the stability of the test results, we repeated the above analysis based on 3 sets from the TCGA database, GSE4412 and GSE43378 sets from the GEO database, and CGGA set, respectively.

As one of the most popular machine learning algorithms for regression and classification tasks [32], the random forest (RF) algorithm is often used to determine the importance of eigengenes [33]. To further assess the relative importance of the eight PR-DE-ERSGs used to build the model in glioma, we ran the RF algorithm. The RF algorithm was run based on the minimum point of cross-validation error to further rank the importance of the 8 PR-DE-ERSGs. This step was performed synchronously in the TCGA, GSE108474, and GSE4290 cohorts. In addition, we also plotted ROC curves based on 8 PR-DE-ERSGs expression of glioma and normal samples from the TCGA dataset to evaluate their diagnostic value in glioma.

### 2.6. Deep Validation of Model's Performance and Stability

The heatmap visually shows the clinical features matched to the risk score of each sample from TCGA. We observed differences in risk scores across subgroups of different clinical characteristics. Kaplan–Meier survival curves further tested the model's ability to distinguish prognosis within each clinical subgroup.

We attempted to obtain glioma and normal brain tissues' immunohistochemical (IHC) staining images of modeled 8 PR-DE-ERSGs from the Human Protein Atlas database. These images were used to compare further the expression differences of the 8 PR-DE-ERSGs at the protein expression level to verify the stability of the model.

Unfortunately, IHC staining images still failed to confirm the differential expression of BMP2, DRAM1, NUP107, and SCG5 between gliomas and normal tissues. Therefore, we further carried out Quantitative Real-time PCR (QRT-PCR) experiments to detect the mRNA expression levels of these four genes.

After obtaining the approval from the Ethics Committee of Shangrao People's Hospital (2022-04-041) and patients' informed consent, we obtained 6 matched pairs of gliomas and adjacent paracancerous tissues from the hospital. All primer sequences were listed in the Supplementary Table 2. Total RNA was isolated from tissues using the TransZol Up Plus RNA Kit (TRANS, Beijing, China). According to the manufacturer's instructions, cDNA was synthesized by using EasyScript First-Strand cDNA Synthesis SuperMix (TRANS, Beijing, China). QRT-PCR was performed by using PerfectStart® Green qPCR SuperMix (TRANS, Beijing, China) on the Roche Light Cycler 96 Real-time Fluorescent Quantitative PCR System. The detection values of 4 genes were normalized to the *β*-actin gene with the 2^−ΔΔCt^ method. After calculating the relative mRNA expression values of the four genes, we compared their differences between glioma and normal paracancerous tissues using paired *t*-test.

### 2.7. Enrichment Analysis for Biological Functions and Pathways Related to Different Risk Groups

Gene Set Enrichment Analysis (GSEA) was utilized to perform Kyoto encyclopedia of genes and genomes (KEGG) and Gene ontology (GO) analyses to screen the signal pathways and biological functions involved in the different risk groups based on expression matrix data [34]. The R package “clusterProfiler” and gene sets “c2.cp.kegg.v7.4.symbols.gmt” and “c5.go.v7.4.symbols.gmt” were applied to run enrichment analysis [34].

### 2.8. Exploring the Potential Role of Tumor Immunosuppressive Environment/Immune Infiltrates Types

After calculating the overall immune and stromal cell scores for each sample using the R package “estimate,” we further quantified 16 immune cells and 13 immune function scores by single-sample gene set enrichment analysis (ssGSEA) based on R packages “GSEABase” and “gsva” [35, 36]. Correlation analysis was run between overall immune cell/stromal cell/16 immune cell/13 immune function scores and risk scores to explore potential relationships between risk scores and immune microenvironment. As reported previously, six types of immune infiltration were identified in human tumors, which corresponded from tumor-promoting to tumor-suppressing, respectively, namely, C1 (wound healing), C2 (INF-g dominant), C3 (inflammatory), C4 (lymphocyte depleted), C5 (immunologically quiet), and C6 (TGF-b dominant) [37]. Heatmaps showed differences in the distribution of different immune infiltrating subtypes between high- and low-risk groups. Boxplots were used to further visualize differences in risk scores between different immune infiltrating subtypes.

### 2.9. Exploring the Potential Role of Cell Stemness

The acquisition of progenitor-like, stem-cell-like features is strongly connected with cancer progression. Stemness, the attribute of self-renewal and differentiation from the cell of origin, can be described by two stemness indexes: a gene expression-based stemness index (mRNAsi) and a DNA methylation-based stemness index (mDNAsi), which are calculated by one-class logistic regression machine learning algorithm (OCLR) training on stem cell (ESC, embryonic stemcell; iPSC, induced pluripotent stem cell) classes and their differentiated ecto-, meso-, and endoderm progenitors [38]. Malta et al. calculated the mDNAsi for each TCGA sample based on OCLR-based transcriptomic and epigenetic signatures [38]. Correlation and difference analyses were again run to explore the potential association of mDNAsi with risk score/various clinical characteristics.

### 2.10. Exploring the Potential Role of HEY2-Related ceRNA Regulatory Network

Gene names of miRNAs for RNA sequencing data from the TCGA database (530 gliomas and 5 adjacent normal tissues) were matched with annotation files of mature miRNAs obtained from miRbase. Considering the critical role of hairy and enhancer of split-related with YRPW motif protein 2 (HEY2) in glioma, we predicted miRNAs and lncRNAs that may regulate HEY2 expression.

To improve the accuracy of the prediction results, we selected miRNAs that appeared more than 3 times in several target gene prediction programs of starBase. The coexpression network between all predicted miRNAs and HEY2 was plotted by Cytoscape (v3.7.2). TCGA data was used to further screen miRNAs with significant roles in glioma. The correlation analysis between miRNAs and HEY2, miRNAs difference analysis between glioma and normal brain tissue, and the Kaplan–Meier survival analysis of miRNAs were performed under the corresponding filtering conditions (correlation analysis: correlation coefficient < −0.2, *p* < 0.001; difference analysis: |log 2FC| > 1 and *p* < 0.05; Kaplan–Meier survival analysis: *p* < 0.05). Eventually, hsa-miR-369-3p and hsa-miR-181a-5p were identified as candidate miRNAs upstream of HEY2.

Afterward, we again predicted potential lncRNAs upstream of hsa-miR-369-3p and hsa-miR-181a-5p, respectively. Similar methods and filtering conditions were used to screen candidate lncRNAs, respectively. Only hsa-miR-369-3p obtained candidate lncRNAs. Cytoscape was again used to map the regulatory network composed of 5 candidate lncRNAs, hsa-miR-369-3p, and HEY2. We again ran correlation analysis between lncRNAs and hsa-miR-369-3p (correlation coefficient < −0.35 and *p* < 0.001), correlation analysis between lncRNAs and HEY2 (correlation coefficient < −0.15 and *p* < 0.001) to filter lncRNAs as the finalized lncRNAs deeply. Two finalized lncRNAs were highlighted in yellow in regulatory networks.

### 2.11. Exploring the Potential Role of Mutation

After detecting and counting somatic gene mutation data in glioma samples in MAF files from the TCGA database, waterfall plots were used to visualize the corresponding results. The correlation analysis between risk score and tumor mutation burden (TMB), the difference analysis of TMB between different risk groups, and the Kaplan–Meier survival analysis of TMB were all used to explore the potential relationship between the model and TMB.

The statistical results show that IDH1/TP53 mutation, the most frequently mutated gene in glioma, may play an indispensable role in glioma. Therefore, after obtaining the effect of IDH1/TP53 mutation on prognosis, we further compared the difference in the risk score/8 PR-DE-ERSGs expression between IDH1/TP53 mutation and wild groups. Considering the predictive value of CD274 in immunotherapy effect, we also compared the CD274 expression difference between the two groups to explore the guiding value of IDH1/TP53 mutation in clinical treatment.

### 2.12. Evaluation of the Guiding Value of the Model in Therapy

At present, chemotherapy and immunotherapy are still the main drug treatment methods for glioma patients [39, 40]. Immunophenoscore (IPS) from The Cancer Immunome Atlas (TCIA) database and Tumor Immune Dysfunction and Exclusion (TIDE) indexes from the TIDE online website have previously been used as predictors of the immunotherapy efficacy [41, 42]. A lower TIDE score and higher IPS represent a better patient response to immunotherapy [41–43]. Therefore, we obtained 3 kinds of IPSs and TIDE/MSI/T cell dysfunction/T cell exclusion scores from these two websites as indicators of immunotherapy's effect. Correlations between 3 IPSs and 8 PR-DE-ERSGs expressions, and correlations between TIDE indexes and 8 PR-DE-ERSGs expressions were analyzed to explore the model's guiding value in immunotherapy efficacy.

The NCI-60 database containing 60 cancer cell lines from 9 different types of tumors was accessed through the CellMiner interface [35]. Then, the association between 8 PR-DE-ERSGs and 263 drugs approved by the FDA or in clinical trials was examined by correlation analyses, respectively.

### 2.13. Extending the Model's Clinical Value

To construct a more accurate comprehensive nomogram to predict the survival probability of glioma patients, we used independent prognostic factors obtained by Cox regression analysis. The calibration curve and ROC curve at 1, 2, and 3 years were used for performance testing of this tool.

Recent studies have shown that immune checkpoint inhibitors (ICIs), N6-methyladenosine (m6A), and multidrug resistance-related genes play an important role in the diagnosis and therapy of gliomas [44–46]. The correlation between the expression levels of 46 ICIs/12 m6A/2 multidrug resistance-related genes and risk scores, and the expression differences of these genes between different risk groups were analyzed to further explore the model's extended clinical value.

### 2.14. Statistical Analysis

This analysis was performed primarily in the R programming language (version 4.0.3) based on numerous packages including “limma,” “WGCNA,” “ComplexHeatmap,” “GSEABase,” “ggExtra,” “maftools,” “regplot,” and “ggplot2.” In data comparison between/among groups, student's *t*-test/chi-square test or nonparametric test/fisher's exact test was used when appropriate. Unless otherwise stated, statistical significance was set at *p* < 0.05.

## 3. Results

### 3.1. Recruitment of Glioma Samples for Obtaining DE-ERSGs

**Figure 2:**
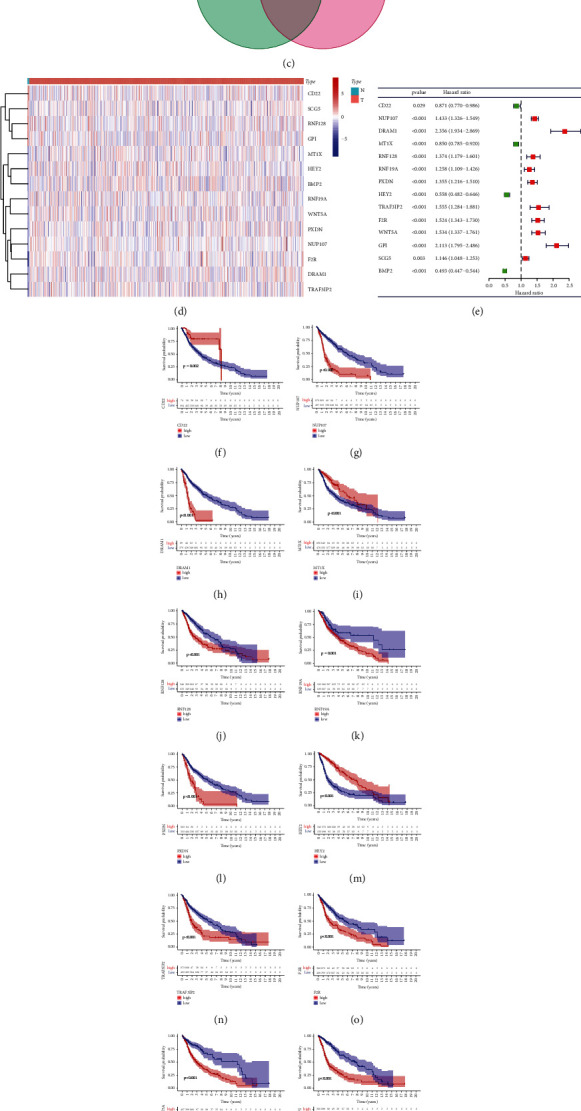
Screening PR-DE-ERSGs. (a) Acquisition of common DE-ERSGs. (b) Acquisition of common DE-ERSGs strongly related to glioma. (c) Acquisition of common PR-DE-ERSGs. (d) The expression levels of 14 common PR-DE-ERSGs in glioma and normal brain tissues. (e) Univariate Cox regression analysis results of 14 common PR-DE-ERSGs. (f)–(s) Kaplan–Meier survival analysis of 14 common PR-DE-ERSGs.

Supplementary Figures 1A and 1B show 1438 DE-ERSGs (up-regulated: 785; down-regulated: 653) are identified from the TCGA dataset based on the 5828 ERSGs' RNA sequencing data. Similarly, there are 2828 DE-ERSGs (up-regulated: 1507; down-regulated: 1321; Supplementary Figures 2A and 2B) extracted from the GSE108474 dataset and 3375 DE-ERSGs (up-regulated: 1886; down-regulated: 1489; Supplementary Figures 3A and 3B) extracted from the GSE4290 dataset. Five hundred eighty-four common DE-ERSGs were obtained by intersecting DE-ERSGs from these 3 datasets and ERSGs from the remaining 3 datasets (Figure 2(a)).

### 3.2. Screening for DE-ERSGs Strongly Related to Glioma

**Figure 3:**
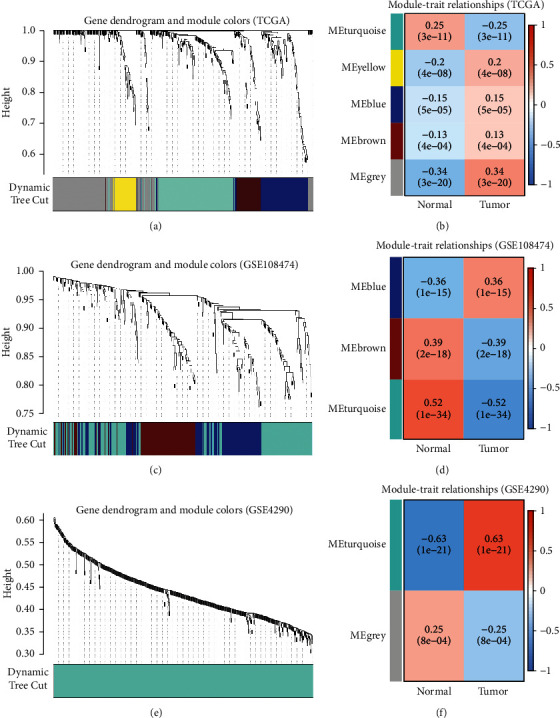
The process of identifying gene modules strongly related to glioma by WGCNA based on TCGA, GSE108474, and GSE4290 datasets, respectively. (a), (c), and (e) The dynamic pruning trees after merging similar modules, respectively. (b), (d), and (f) The heatmaps reflecting the correlation between modules and gliomas, respectively. The correlation coefficients and corresponding *p*-values for each module are shown in the boxes.

After setting the best soft power *β*, the dynamic pruning trees merged the similar modules and matched each gene with the corresponding gene module based on TCGA, GSE108474, and GSE4290 datasets, respectively (Figures 3(a), 3(c), and 3(e)). The heatmaps in Figures 3(b), 3(d), and 3(f) show the correlation between gene modules identified by WGCNA and glioma status based on these three datasets, respectively. We extracted 24 common DE-ERSGs strongly related to glioma from TCGA grey module, GSE108474 blue module, and GSE4290 turquoise module using Venn diagram (Figure 2(b)).

### 3.3. Screening PR-DE-ERSGs for Prognostic Model's Construction Based on LASSO Regression

Fourteen common PR-DE-ERSGs were obtained from 20 PR-DE-ERSGs in the TCGA dataset and 17 PR-DE-ERSGs in the CGGA dataset (Figure 2(c)). The expression levels of these 14 genes in glioma and normal brain tissues are shown in Figure 2(d). Except for CD22, MTX1, BMP2, and HEY2 (HR < 1), the remaining 10 genes (HR > 1) were identified as risk factors in the TCGA dataset, as shown in the forest plot (Figure 2(e)) and Kaplan–Meier survival curves (Figures 2(f)–2(s)). It can be seen that random grouping did not cause differences in the distribution of data between groups (Table 1, *p* > 0.05). By setting the optimal value of *λ* by the LASSO regression analysis (Supplementary Figures 4A and 4B), we acquired 8 PR-DE-ERSGs to construct the model, eventually. The corresponding coefficients of the 8 PR-DE-ERSGs are detailed in Supplementary Table 1.

### 3.4. Validation of Differential Expression of 8 Genes by External Data

Supplementary Figures 5A–5H show the differential analysis results of 8 genes from the GEPIA database. Consistent with our previous findings, the expressions of NUP107, DRAM1, RNF19A, PXDN, HEY2, F2R, and BMP2 were up-regulated in gliomas (glioblastoma multiforme + lower grade glioma). The stable expression trends of these genes all support the good stability of our model.

### 3.5. Model's Performance and Stability Testing

**Figure 4:**
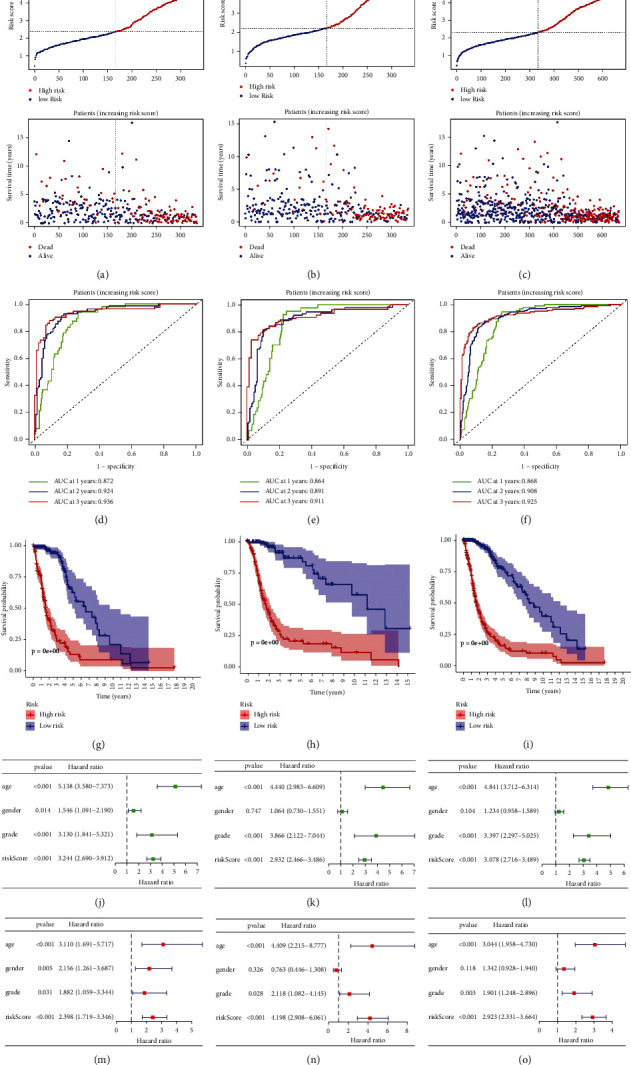
Model performance testing based on training dataset (a), (d), (g), (j), and (m), test dataset (b), (e), (h), (k), and (n), whole set (c), (f), (i), (l), and (o). (a)–(c) Changes in sample's survival with increasing risk score. (d)–(f) ROC curves reflecting model's performance. (g)–(i) Kaplan–Meier survival curves reflecting model's prognostic discrimination performance. (j)–(l) Results of univariate Cox regression analysis to determine influencing factors of prognosis. (m)–(o) Results of multivariate Cox regression analysis to determine independent influencing factors of prognosis.

Samples from all three sets of TCGA exhibited worse prognosis as the risk score increased (Figures 4(a)–4(c)). This conclusion was also supported in three external datasets samples (Supplementary Figures 6A–6C). Figures 4(d)–4(f) show that our model achieved an area under the curve (AUC) value of >0.85 in three sets of TCGA data. Surprisingly, such excellent performance can still stand in the test of external datasets (all AUC values > 0.65, Supplementary Figures 6D–6F). Kaplan–Meier survival curves show significant differences in survival probability between different risk groups. This conclusion is applicable to the corresponding tests based on all internal and external datasets (Figures 4(g)–4(i) and Supplementary Figures 6G–6I). Both univariate and multivariate analyses based on 6 sets all confirmed that risk score was a prognostic factor for patients with glioma (Figures 4(j)–4(o) and Supplementary Figures 6J–6O). Together, the above results support the excellent performance of our model in predicting the prognosis of glioma patients.

The effect of the number of decision trees on the error rate from three datasets is shown in Supplementary Figures 7A–7C. From the 3 datasets, we observed that the importance scores of the 8 ERSGs were relatively high (all importance scores > 0.5), which suggested that these genes played relatively important roles in the model (Supplementary Figures 7D–7F). The diagnostic ROC curves of the genes used for modeling all verified their high diagnostic value in glioma (all AUC > 0.85, Supplementary Figures 7G–7M). These results support the stability of the model constructed by these eight genes.

### 3.6. Deep Validation of Model Performance and Stability

**Figure 5:**
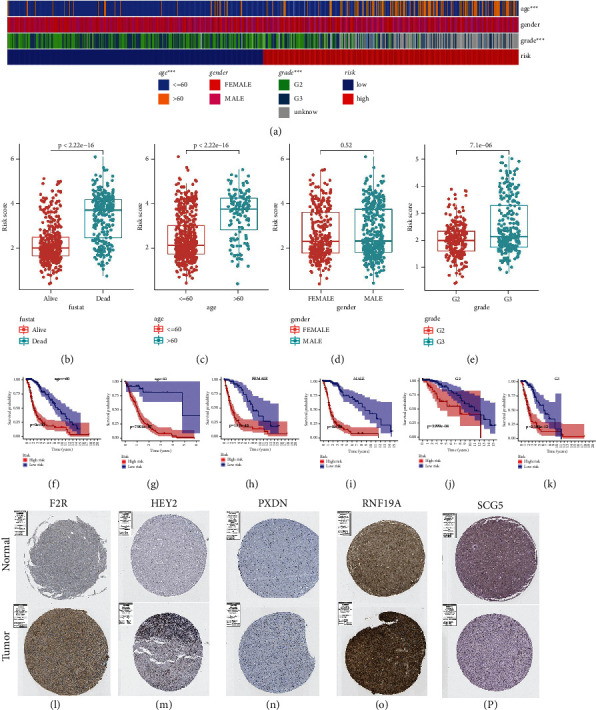
Deep validation of model's performance and stability based on TCGA dataset. (a) Heatmap showing clinical features matched to each sample's risk score. ^*∗∗∗*^*p* < 0.001. (b)–(e) Risk score differences between subgroups with different clinical features. (f)–(k) Kaplan–Meier survival analysis reflecting model's prognostic discrimination performance in each clinical feature subgroup. (l)–(p) IHC staining images of F2R, HEY2, PXDN, RNF19A, and SCG5 in normal brain and glioma tissues, respectively.

The heatmap visually shows 3 clinical features matched to the risk score of each sample from TCGA (Figure 5(a)). Additionally, the following Wilcoxon signed-rank test further provided many exciting results with clinical value. The risk scores between survival status, age, and grade subtypes show dramatical differences (Figures 5(b), 5(c), and 5(e)), demonstrating the close association between survival status/age/grade and risk score. It is worth pointing out that patients in the dead group, age ༞60 group, and G3 group were observed to have a higher risk score. However, gender characteristics did not show a similar conclusion (Figure 5(d)). More importantly, there were also significant differences in survival probability between samples from different risk groups within each clinical feature subgroup (Figures 5(f)–5(k)), indicating the guiding significance of risk score for prognosis.

We only obtained IHC staining images reflecting the protein expression of five PR-DE-ERSGs (F2R, HEY2, PXDN, RNF19A and SCG5) (Figures 5(l)–5(p)). As shown in the stained images, the four PR-DE-ERSGs (F2R, HEY2, PXDN, and RNF19A) exhibited differences in protein expression levels between glioma and normal brain tissues, consistent with their differential expression results based on the TCGA dataset (Figures 5(l)–5(o)). Unfortunately, the image of SCG5 fails to support the analysis results based on the TCGA dataset (Figure 5(p)).

**Figure 6:**
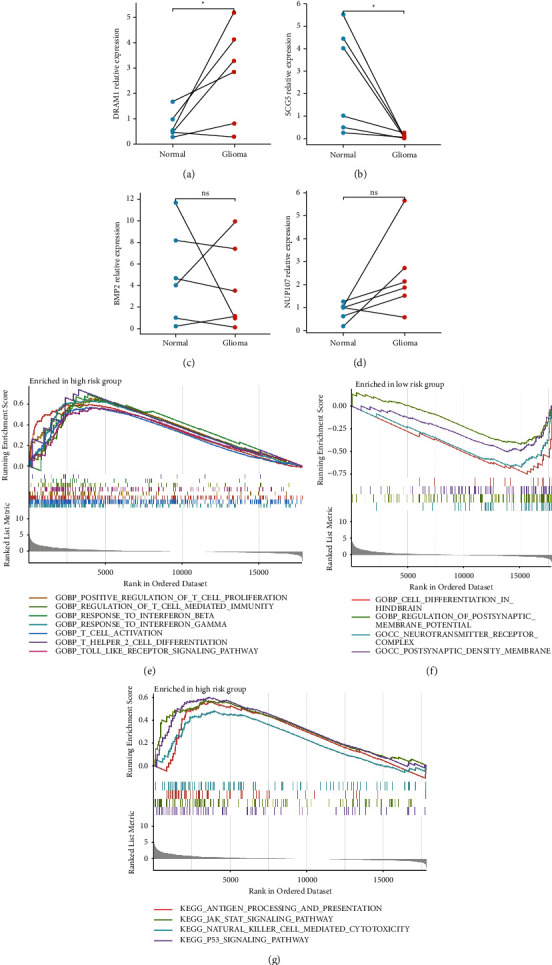
The differential analysis of 4 modeled genes' relative mRNA expression detected by qRT-PCR and enrichment analysis results of different risk groups. (a) DRAM1. (b) SCG5. (c) BMP2. (d) NUP107. (e) GO enrichment analysis results of the high-risk group. (f) GO enrichment analysis results of the low-risk group. (g) KEGG enrichment analysis results of the high-risk group.

Figures 6(a) and 6(b)showed the significantly higher DRAM1 and lower SCG5 relative mRNA expression in gliomas. But we still failed to verify the significant differential relative mRNA expression of BMP2 and NUP107 between gliomas and adjacent normal tissues (Figures 6(c) and 6(d)).

### 3.7. Enrichment Analysis for Biological Functions and Pathways Related to Different Risk Groups

To explore the relationship between different risk groups and biological pathways, GSEA was utilized to catalogue biological pathways modulated by risk groups. In the high-risk group, most of the GO analysis results and KEGG analysis results were immune-related functions (positive regulation of T cell proliferation, regulation of T cell mediated immunity, response to interferon-beta, T cell activation, T helper 2 cell differentiation, toll-like receptor signaling pathway) and pathways (antigen processing and presentation, JAK-STAT signaling pathway, natural killer cell mediated cytotoxicity, p53 signaling pathway) (Figures 6(e) and 6(g)). Likewise, the biological functions in the low-risk group are shown in Figure 6(f) (including cell differentiation in hindbrain, regulation of postsynaptic membrane potential, neurotransmitter receptor complex, and postsynaptic density membrane).

### 3.8. Exploring the Potential Role of Immune Infiltrates Types and Cell Stemness

**Figure 7:**
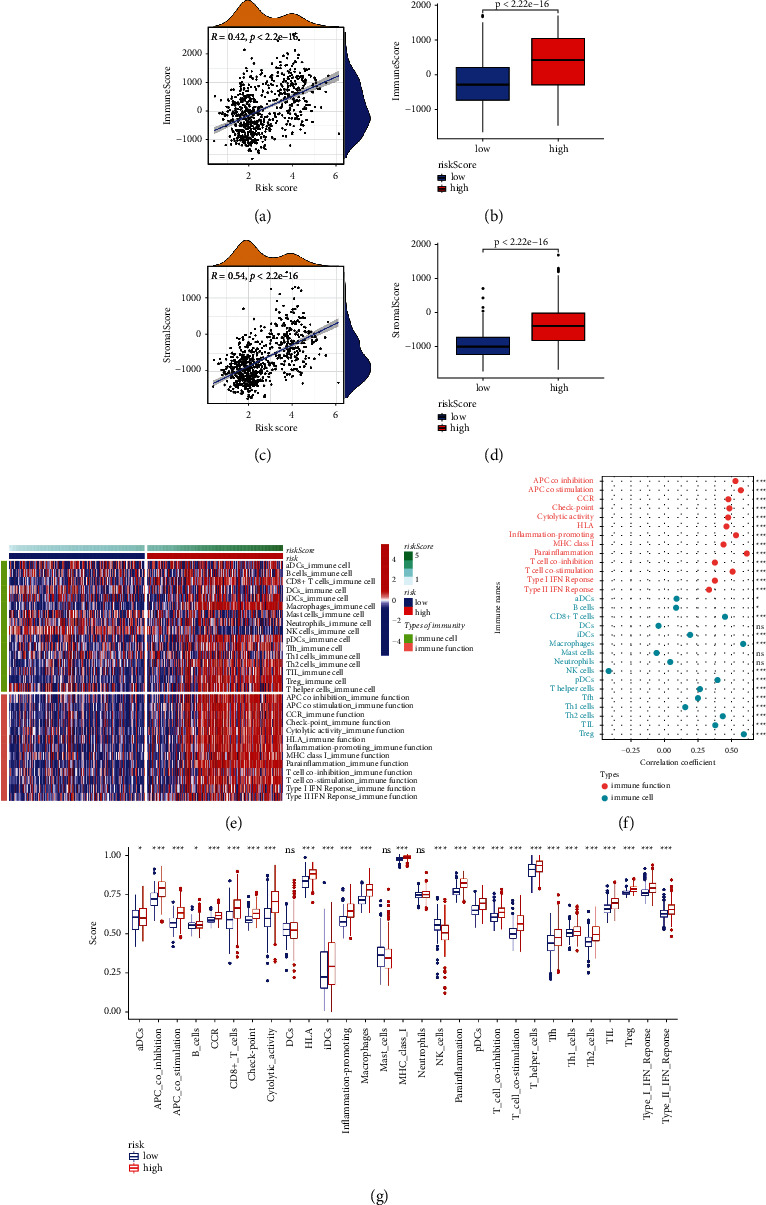
Exploratory results related to tumor immunosuppressive environment. (a), (c), and (f) Correlation analysis results between risk score and overall immune cell/overall stromal cell/16 immune cells and 13 immune functions scores. (b), (d), and (g) Overall immune cell/overall stromal cell/16 immune cells and 13 immune functions scores difference between different risk groups. (e) The distribution of 16 immune cells and 13 immune functions scores as risk scores increased. ns: no significance; ^*∗*^*p* < 0.05; ^*∗∗*^*p* < 0.01; ^*∗∗∗*^*p* < 0.001.

Both correlation and difference analysis results supported a positive association between the overall immune/stromal cell score and the risk score (Figures 7(a)–7(d)). The distribution of 16 immune cells and 13 immune function scores as risk scores increased preliminarily showed their positive association with risk score (Figure 7(e)). Figures 7(f) and 7(g) further show the specific association of each immune cell/function score with the risk score. Notably, regulatory T (Treg) cells, CD8+ T cells, immature dendritic cells (iDCs), T helper 2 (Th2) cells, and macrophages were all positively associated with risk scores, and their higher scores in the high-risk group were also observed.

### 3.9. Exploring the Potential Role of Tumor Immunosuppressive Environment

**Figure 8:**
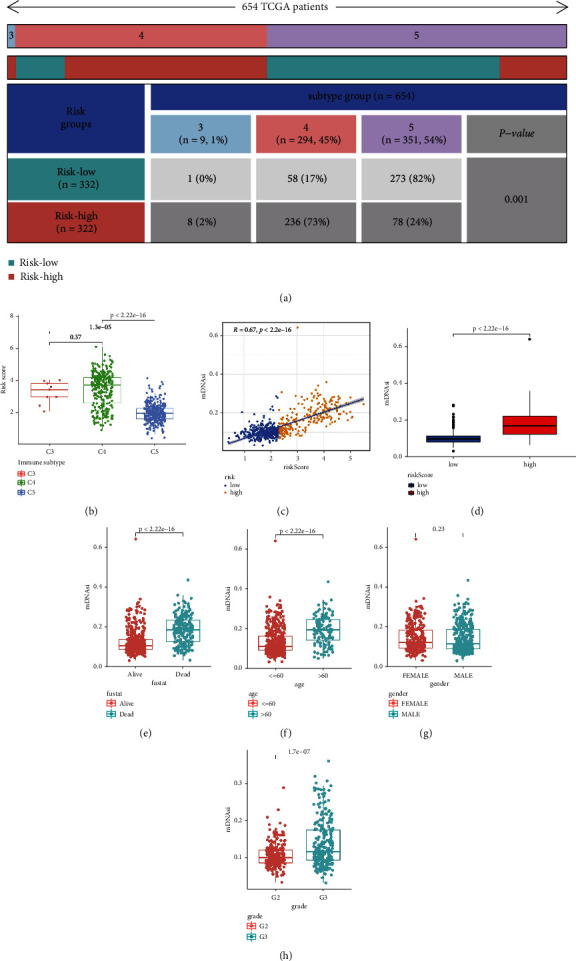
Exploratory results of immune infiltration subtypes and cell stemness analysis. (a) The heatmap shows the proportion of C3, C4, and C5 subtypes in different risk groups. (b) Pairwise comparison of risk scores among C3, C4, and C5 subtypes. (c) Correlation analysis result between mDNAsi and risk score. (d) mDNAsi difference between the high-risk and the low-risk groups. (e)–(h) Boxplots visualized mDNAsi differences between different clinical subgroups.

The heatmap showed significant differences in the number of samples of C3, C4, and C5 immune infiltrating subtypes between different risk groups (Figure 8(a)). Higher risk scores in the C3/C4 subgroup and lower risk scores in the C5 subgroup further confirmed this distribution (Figure 8(b)). The above results indicated that the C5 subtype was dominant in the low-risk group, and the C3 and C4 subtypes were dominant in the high-risk group.

Figures 8(c) and 8(d) show that mDNAsi is positively correlated with the risk score. We also explored the relationship between mDNAsi and four clinical features (survival state, age, gender, and grade). We found that mDNAsi had significantly high scores in the dead (Figure 8(e)), age > 60 (Figure 8(f)) and G3 grade (Figure 8(h)) populations. However, no significant association between gender and mDNAsi was found (Figure 8(g)). The above results imply the role of cell stemness in promoting glioma's progression.

### 3.10. Exploring the Potential Role of HEY2-Related ceRNA Regulatory Network

**Figure 9:**
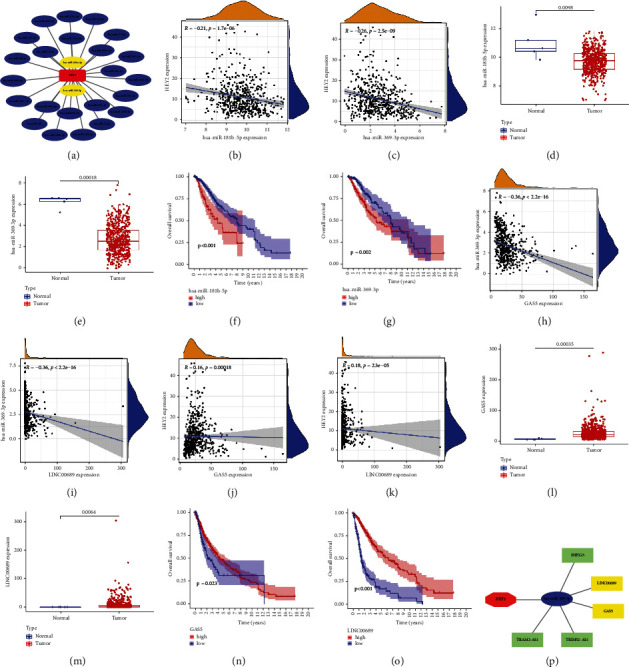
Exploratory results of HEY2-related ceRNA regulatory network. (a) The potential network of 25 miRNAs regulating HEY2 expression. (b) and (c) Correlation analysis result of HEY2 with miR-181a-5p/miR-369-3p, respectively. (d) and (e) Difference analysis result of the miR-181a-5p/miR-369-3p expression between normal and tumor tissue, respectively. (f) and (g) The effects of miR-181a-5p/miR-369-3p on prognosis of glioma patients, respectively. (h) and (i) Correlation analysis results of miR-369-3p with GAS5/LINC00689, respectively. (j) and (k) Correlation analysis results of HEY2 with GAS5/LINC00689, respectively. (l) and (m) Difference analysis result of the GAS5/LINC00689 expression between normal and tumor tissue, respectively. (n) and (o) The effects of GAS5/LINC00689 on prognosis of glioma patients, respectively. (p) A ceRNA regulatory network composed of 5 candidate lncRNAs, miR-369-3p and HEY2 (yellow box: correlation coefficient < −0.35, green box: correlation coefficient < −0.2).

Of all predicted miRNAs, only 25 miRNAs potentially regulating HEY2 exceeded three times in Starbase database prediction procedures. The corresponding coexpression network is shown in Figure 9(a). The mechanism whereby miRNAs regulate the expression of target genes elucidates that the potential miRNAs should be inversely correlated with HEY2 expression [47]. The negative correlation between miR-181b-5p/miR-369-3p and HEY2 expression level and the significantly lower expression of these two genes in glioma both support their negative regulation of HEY2 expression (Figures 9(b)–9(e)). The lower survival probability observed in the high expression group of these two miRNAs further confirms their essential roles in glioma progression (Figures 9(f) and 9(g)). The above analysis results well supported the roles of miR-181a-5p and miR-369-3p as upstream candidate miRNAs in regulating HEY2 expression.

The prediction procedures predicted 122/54 lncRNAs might regulate the expression of miR-181a-5p/miR-369-3p, respectively. Stringent filtering conditions ultimately provided only two final candidate lncRNAs for miR-369-3p. Eventually, the entire coexpression network is visualized in Figure 9(p). Corresponding correlation and differential analysis results further confirmed the inverse regulatory relationship between these two lncRNAs (LINC00689 and GAS5) and miR-369-3p in glioma (Figures 9(h)–9(m)). These two lncRNAs also showed a positive effect on the prognosis of glioma patients (Figures 9(n) and 9(o)).

### 3.11. Exploring the Potential Role of Mutation

**Figure 10:**
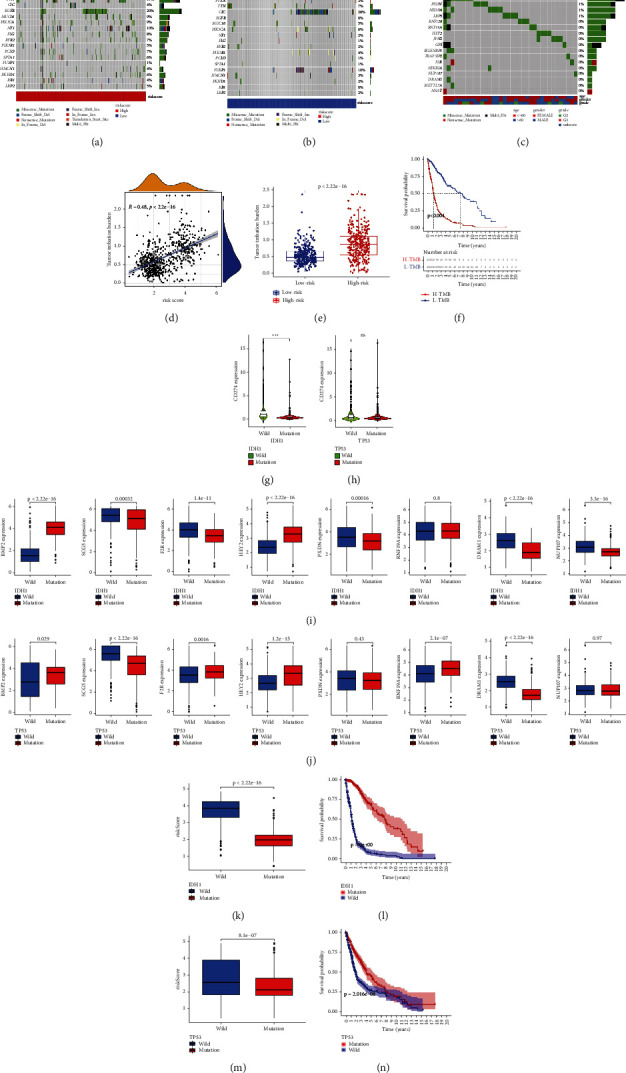
Exploratory results of mutation. (a) and (b) An overview of the mutation distribution in the samples of different risk groups of the top 20 genes with mutation frequency, respectively. (c) A mutation distribution overview of 17 mutated DE-ERSGs strongly related to glioma. (d) Correlation analysis result between risk score and TMB. (e) Difference analysis result of TMB between different risk groups. (f) Kaplan–Meier survival analysis showed the effect of TMB on prognosis. (g) and (i) CD274/8 PR-DE-ERSGs expression difference analysis between IDH1 mutant and wild groups. (h) and (j) CD274/8 PR-DE-ERSGs expression difference analysis between TP53 mutant and TP53 wild groups. (k) and (l) Risk score and survival difference between IDH1 mutant and wild groups. (m) and (n) Risk score and survival difference between TP53 mutant and TP53 wild groups.

The waterfall charts provide an overview of the mutation distribution of the top 20 genes with mutation frequency in the samples of different risk groups (Figures 10(a) and 10(b)). TP53 and IDH1 are the two genes with the highest mutation frequency in those two charts. In addition, 17 of 24 DE-ERSGs strongly related to glioma were mutated (Figure 10(c)). Correlation and difference analysis both supported the close association between TMB and the model (Figures 10(d) and 10(e)). Clearly, TMB has a negative prognostic effect on gliomas (Figure 10(f)). Figures 10(i)–10(k) and 10(m) reveal a strong association between TP53/IDH1 mutation and 8 PR-DE-ERSGs/risk score, suggesting cooperative roles of TP53/IDH1 mutation and ERS in glioma. Figures 10(l) and 10(n) show the positive effect of TP53/IDH1 mutation on the prognosis of glioma patients. Lower CD274 expression in the TP53/IDH1 mutation group revealed the predictive value of TP53/IDH1 mutation in response to immunotherapy (Figures 10(g) and 10(h)).

### 3.12. Evaluation of the Guiding Value of the Model in Therapy

**Figure 11:**
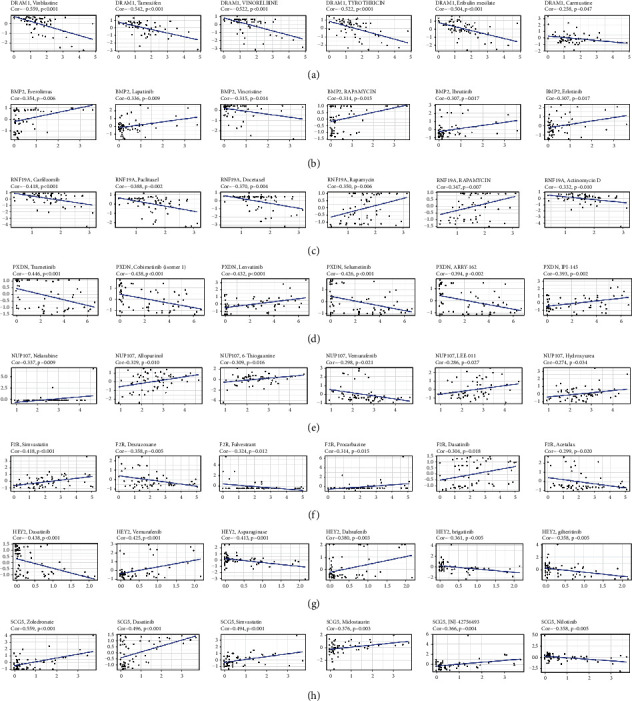
Correlation analysis results between the top six drugs most closely related to the 8 PR-DE-ERSGs expression and 8 PR-DE-ERSGs, respectively. (a) DRAM1. (b) BMP2. (c) RNF19A. (d) PXDN. (e) NUP107. (f) F2R. (g) HEY2. (h) SCG5.

**Figure 12:**
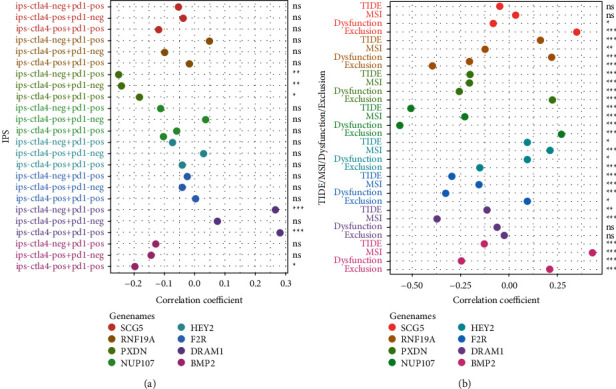
Evaluation of the correlation between 8 PR-DE-ERSGs and immunotherapy response. (a) Correlation analysis result between 3 types of IPS and 8 PR-DE-ERSGs expression. (b) Correlation analysis result between TIDE/MSI/T cell dysfunction/T cell exclusion score and 8 PR-DE-ERSGs expression. ns: meaningless; ^*∗*^*p* < 0.05; ^*∗∗*^*p* < 0.01; ^*∗∗∗*^*p* < 0.001. *P* < 0.05 is considered to be statistically significant.

Figures 11(a)–11(h) show the corresponding results for the top six most strongly correlated with 8 PR-DE-ERSGs expression among 263 FDA approved or clinically tested drugs. It is worth mentioning that we observed an inverse correlation between DRAM1 expression and carmustine (a chemotherapeutic drug recommended by the latest National Comprehensive Cancer Network (NCCN) guidelines for the treatment of glioma) (Figure 11(a)). This suggests that the 8 PR-DE-ERSGs in the model may serve as guide markers for the rational use of these drugs. Combining the correlation results, we found that the expressions of PXDN, DRAM1, and BMP2 were all closely correlated with IPSs and TIDE scores, implying the guiding value of these genes in immunotherapy strategies (Figures 12(a) and 12(b)).

### 3.13. Extending the Model's Clinical Value

**Figure 13:**
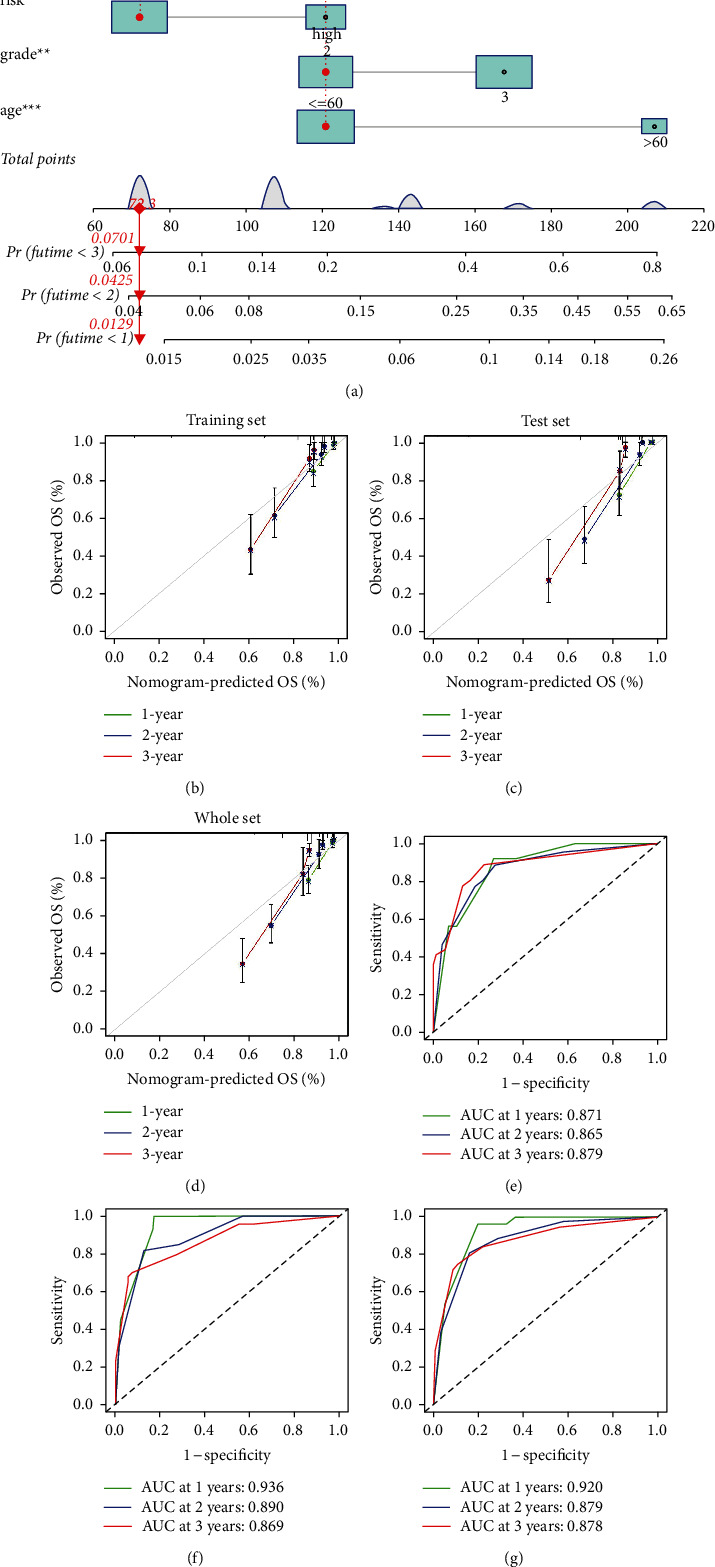
Construction of a nomogram that effectively extends the clinical value of the model. (a) A novel nomogram to accurately predict the 1-, 2- and 3-year survival probability of glioma patients. (b)–(d) Internal calibration curves of 1, 2 and 3 years based on three sets, respectively. Higher degree of fitting with the grey line represents higher accuracy of the Nomogram prediction. (e)–(g) ROC curves of 1, 2 and 3 years based on three sets, respectively. AUC > 0.5 is considered to have a predictive value.

To expand the clinical utility scope of the model, we developed a novel nomogram to facilitate the prediction of the 1-, 2-, and 3-year survival probability of glioma patients (Figure 13(a)). Our nomogram considers the combined effects of composite factors (risk group, age, and grade) on prognosis. The calibration curves demonstrate the excellent internal consistency between the predictions made by our nomogram and the actual outcomes (Figures 13(b)–13(d)). Also, ROC curves show high AUC values in three sets of TCGA (Figures 13(e)–13(g)).

Interestingly, with the exception of VTCN1, TMIGD2, CD200, HHLA2, and ADORA2A, most ICIs related gene expression showed positively correlated with the risk score (Supplementary Figure 8A). In addition, half of the m6A-related genes demonstrated a positive correlation with the risk score (Supplementary Figures 8A and 8C). The following difference analysis results shown in Supplementary Figures 8B and 8C further support the above conclusions.

After analyzing the correlation between multidrug resistance-related genes and the prognosis model, we found that ABCC1 and ABCC3 gene expressions were significantly positively correlated with the risk score (Supplementary Figures 8D and 8F). And both ABCC1 and ABCC3 were highly expressed in the high-risk group (Supplementary Figures 8E and 8G).

## 4. Discussion

WGCNA, a systems biology method for describing the correlation patterns among genes across microarray samples, can be used for finding clusters (modules) of highly correlated genes and relating modules to one another and to external sample traits [48]. WGCNA is widely used to identify tumor candidate biomarkers and explore the mechanism, such as gastric cancer [49], bladder cancer [50], gastric adenocarcinoma [51], and breast cancer [52]. This study utilized WGCNA and univariate Cox regression to screen out 8 PR-DE-ERSGs highly correlated with glioma to establish a prognostic model. We also established a nomogram by combining the prognosis model with clinicopathological factors to predict survival in glioma patients. In repeated validation tests, both the prognostic model and nomogram demonstrated excellent prognostic performance and substantial clinical value. The strong association between risk score and immune cells/functions, cell stemness, and IDH1/TP53 mutation further elucidates the important role of ERS in glioma progression and treatment. The TCGA data further confirmed the important regulatory value of the predicted HEY2/miR-369-3p/lncRNAs networks in glioma.

To better understand the potential molecular mechanism of our prognostic model, we have expanded on these genes. As an essential component of the nuclear pore complex, Nucleoporin 107 **(**NUP107) may contribute to the regulation of cell fate in aged and transformed cells by modulating nuclear trafficking of signal based on undifferentiated oligodendroglioma cells in vitro [53]. Damage Regulated Autophagy Modulator 1 (DRAM1), a p53 target gene encoding a lysosomal protein that induces macroautophagy, plays a central role in p53/TP53-mediated apoptosis [54]. Galavotti et al. found that high levels of DRAM1 were associated with shorter overall survival in GBM patients, which is consistent with our findings [55]. Ring Finger Protein 19A (RNF19A) encodes a protein that belongs to the ring fingers protein family, an E3 ubiquitin ligase [56]. RNF19A decreases p53 expression and its downstream signaling, binds to p53, and promotes its ubiquitination, thereby promoting nonsmall cell lung carcinoma (NSCLC) growth and progression [57]. Peroxidasin (PXDN) encodes a heme-containing peroxidase that is involved in extracellular matrix formation associated with prostate cancer [58]. Overexpression of PXDN is found in ovarian cancer [59], melanoma [60], oral squamous cell carcinoma [61], and prostate cancer [58], associated with poor prognosis. All aforementioned studies support our findings that PXDN is a prognostic risk factor in glioma. Coagulation factor 2 thrombin receptor (F2R), a key component in the thrombosis process, has been demonstrated up-regulated in various cancers and was associated with several protumoral responses, including primary growth, invasion, metastasis, and angiogenesis [62–66]. In glioma, the expression of F2R was up-regulated and predicted poor prognosis. F2R promotes glioma cell proliferation and metastasis under SOX2 and actives the WNT/*β*-catenin signaling pathway [62]. The epigenetic inactivation of secretogranin V (SCG5/SGNE1/7B2) was a frequent early event in glioma formation, resulting in significant downregulation of SCG5/SGNE1/7B2 expression [67]. Bone morphogenetic protein 2 (BMP2), a member of the transforming growth factor-*β* (TGF-*β*) super-family, is one of the main chondrogenic growth factors involved in cartilage regeneration [68]. In the new glioma grading model constructed by Zhou et al., BMP2 shows reliable prognostic value as a protective factor in GBM, consistent with our results [69]. In our study, the results of human tissue-based IHC staining and QRT-PCR experiments well confirmed the biological value of F2R, HEY2, PXDN, RNF19A, SCG5, and DRAM1 in glioma.

Recently, the close functional relationship between ERS and noncoding RNAs (ncRNAs), including miRNAs, lncRNAs, and circRNAs, has been widely reported in cancer development [70]. MiRNAs and lncRNAs directly or indirectly act on UPR pathway molecules to regulate intracellular homeostasis and affect carcinogenic processes, including survival, apoptosis, invasion, metastasis, cancer stem cell characteristics, and the tumor microenvironment [70]. However, few studies focus on ERS-related ceRNA regulatory network in gliomas. After prediction and verification of TCGA data, a ceRNA regulatory network composed of HEY2, miR-369-3p, and GAS5/LINC00689 was screened out.

HEY2, one of the most prominent Notch pathway target genes, encodes a transcription factor [71]. Halani et al. proposed that the loss of the Notch pathway activity and particularly of Hey2 levels were correlated with oligodendroglioma [72]. Moreover, Giachino et al. found that high HEY2 expression correlates with a better prognosis for patients with grades II–III astrocytoma and GBM, further supporting our observation [73]. Previous studies have shown that overexpression of growth arrest-specific transcript 5 (GAS5) inhibits the malignant phenotype of glioma cells, including proliferation, migration, and invasion, acting as a tumor suppressor of human gliomas [74]. GAS5 may blunt the resistance of glioma cells to cisplatin by suppressing excessive autophagy through the activation of mTOR signaling, implying a promising therapeutic strategy against chemoresistance in glioma [75]. However, increased LINC00689 level was associated with poor clinical features and decreased overall survival in glioma patients, and the overexpression of miR-369-3p inhibited the proliferation and migration in glioblastoma cells [76, 77]. Those appear to be different from ours. However, the predominant roles of HEY2, miR-181a-5p, and GAS5 and the cross-talk between multiple ceRNAs may explain our results. Interestingly, miR-369-3p, miR-181a-5p, and LINC00689 were found to be related to glycolysis [77–79]. And the high rate of aerobic glycolysis is a key characteristic of cancer cells, which promotes uncontrolled proliferation [80]. Recently, accumulating studies have illustrated that activated aerobic glycolysis participated in various cellular and clinical activities of glioma, thus influencing the efficacy of radiotherapy and chemotherapy [80]. Taken together, miRNA dysregulation in glioma disables proper tumor suppression, increases glycolytic metabolism, and augments tumor malignancy through multiple effectors and signaling pathways [81].

The majority of immune cell and function scores in the high-risk group further explain the enrichment of numerous immune-related biological processes in the high-risk group. Infiltration of Treg cells is often enriched in tumor tissue, and a high proportion of Treg cells and effector T cells often predicts poorer patient outcomes [82]. It has been suggested that Treg cells in tumor-associated tertiary lymphoid structures can suppress endogenous immune responses against tumors, resulting in poor prognosis [83]. More and more studies have shown that tumor-associated myeloid cells, such as iDCs, can be recruited in the tumor environment and form an organic whole to maintain an immunosuppressive state [84]. The Siglec-9 expressed on this cell can bind to the Mucins1-related STn antigen released by cancer cells and lead to an increase in interleukin-10 and a decrease in the production of IL-12 [85]. This prevents the DC's ability to activate T-helper 1 responses [86]. According to the tissue environment, macrophages can be divided into M1 macrophages with proinflammatory and tumor-suppressive effects and M2 macrophages with immunosuppressive and tumor-promoting effects [87]. While tumor-associated macrophages generally acquire the same phenotype as M2 macrophages, their roles in cancer are often associated with poor prognosis [88]. Kakizaki et al. found that the cells, together with Tregs, play a role in maintaining the tumor microenvironment [89]. In conclusion, the reason for the poor prognosis of the high-risk group but the enrichment of more immune-related processes may be related to the fact that these immune cells can maintain the tumor immunosuppressive environment.

The waterfall plot showed that the two most frequently mutated genes were isocitrate dehydrogenases 1 (IDH1) and Tumor Protein p53 (TP53) in the high-risk and low-risk groups. IDH1 mutation is an early event in glioma development and positively correlates with TP53 mutation [90, 91]. And the co-occurrence of IDH1 mutations and TP53 alterations is widespread in gliomas [92]. An increasing number of reports have shown that gliomas patients with IDH1 mutations have a better prognosis than the one with wild-type IDH1 [93, 94]. IDH1 is increasingly recognized as an independent prognostic marker for gliomas, consistent with our findings [95]. Importantly, Amankulor et al. suggested that differences in immune cell content may contribute to this prognostic difference [96]. By creating mouse models, they found reduced levels of many types of immune cells in IDH1-mutated tumors, such as macrophages, microglia, monocytes, and neutrophils [96]. Also, previous reports suggested that significant infiltration of these immune cells leads to poor prognosis in a variety of cancers [88, 97–99]. Surprisingly, immune cells such as macrophages were also less in the patients of the low-risk group with a better prognosis. Based on the mutual support between these studies and our findings, we speculate that the favorable prognosis of the IDH1 mutant group may also be related to the reduction of immune cells capable of maintaining the tumor immunosuppressive environment.

As the results show, our model exhibited excellent capabilities in predicting the prognosis of patients with glioma and facilitating the optimal selection of clinical treatment strategies. However, the research still has numerous limitations. For example, the difference in protein expression of SCG5 is still not supported by IHC staining images obtained based on experiments. In addition, our research failed to combine basic experiments to verify the study results. Considering the complexity of the ceRNA network, we only selected HEY2 to explore the ceRNA axis to avoid involving a large number of RNA transcription samples that make the detailed study laborious. Despite many shortcomings, our study still provides a rich exploration and in-depth explanation of the underlying mechanism. These may provide new ideas for follow-up research.

## 5. Conclusions

In this study, 8 PR-DE-ERSGs strongly related to glioma were screened out by WGCNA to establish a prognostic model with excellent prognostic performance and important clinical value. The strong association between risk score and immune cells/functions, cell stemness, and IDH1/TP53 mutation further elucidates the important role of ERS in glioma progression and treatment. The TCGA data further confirmed the important regulatory value of the predicted HEY2/miR-369-3p/lncRNAs networks in glioma. All in all, the above results all supported that biological processes related to HEY2-based ceRNA regulatory network, tumor immunosuppressive environment, cell stemness, and IDH1 mutation may be involved in the progression and treatment of glioma.

## Figures and Tables

**Table 1:** Clinical characteristics of glioma samples in training, test, and whole sets from TCGA.

	Whole set (*n* = 668)	Training set (*n* = 334)	Test set (*n* = 334)	*P*
*Gender (%)*				
Male	385 (57.6%)	186 (55.7%)	199 (59.6%)	0.3474
Female	283 (42.4%)	148 (44.3%)	135 (40.4%)	

*Age (year, %)*				
≤60	529 (79.2%)	255 (76.3%)	274 (82.0%)	0.0862
>60	139 (20.8%)	79 (23.7%)	60 (18.0%)	

*Overall survival time (day, %)*				
≤730	407 (60.9%)	200 (59.9%)	207 (62.0%)	0.6342
>730	261 (39.1%)	134 (40.1%)	127 (38.0%)	

*Survival status (%)*				
Alive	414 (62.0%)	197 (59.0%)	217 (65.0%)	0.1299
Dead	254 (38.0%)	137 (41.0%)	117 (35.0%)	

*Grade (%)*				
2	247 (37.0%)	121 (36.2%)	126 (37.7%)	0.9234
3	261 (39.1%)	130 (38.9%)	131 (39.2%)	
Unknown	160 (23.9%)	83 (24.9%)	77 (23.1%)	

**Table 2:** Clinical characteristics of glioma samples in 2 GEO cohorts and CGGA cohort.

	GSE4412 cohort (*n* = 85)	GSE43378 cohort (*n* = 50)	CGGA cohort (*n* = 983)
*Gender (%)*			
Male	32 (37.6%)	34 (68.0%)	405 (41.2%)
Female	53 (62.4%)	16 (32.0%)	578 (58.8%)

*Age (%)*			
≤60	70 (82.4%)	29 (58.0%)	869 (88.4%)
༞60	15 (17.6%)	21 (42.0%)	113 (11.5%)
Unknown	0 (0.0%)	0 (0.0%)	1 (0.1%)

*Survival status*			
OS-days (median, range)	389 (7–2516)	545 (20–3020)	777 (19–4697)
OS-state (alive (%)/dead (%))	26 (30.6%)/59 (69.4%)	8 (16.0%)/42 (84.0%)	387 (39.4%)/596 (60.6%)

*Grade (%)*			
2	0 (0.0%)	5 (10.0%)	280 (28.5%)
3	26 (30.6%)	13 (26.0%)	325 (33.1%)
4	59 (69.4%)	32 (64.0%)	374 (38.0%)
Unknown	0 (0.0%)	0 (0.0%)	4 (0.4%)

## Data Availability

The datasets analyzed in this study came from databases shared publicly. Data can be obtained from CellMiner https://discover.nci.nih.gov/cellminer, CGGA http://www.cgga.org.cn/, GeneCards http://www.genecards.org/, GEO https://www.ncbi.nlm.nih.gov/geo/, HPA http://www.proteinatlas.org/, mirbase https://www.mirbase.org/, StarBase http://starbase.sysu.edu.cn/, TCGA https://cancergenome.nih.gov/, TCIA https://tcia.at/home, TIDE http://tide.dfci.harvard.edu/.

## Supplementary Materials

Supplementary Materials Supplementary Figure 1. Differential expression analysis of ERSGs in the TCGA dataset. (A) Heap map of the differential expression of ERSGs in the TCGA dataset. (B) Volcano plot of DE-ERSGs in the TCGA dataset. (red dots: up-regulated genes, black dots: genes with no significant differences, green dots: down-regulated genes). Supplementary Figure 2. Differential expression analysis of ERSGs in the GSE108474 dataset. (A) Heap map of the differential expression of ERSGs in the GSE108474 set. (B) Volcano plot of DE-ERSGs in the GSE108474 set. (red dots: up-regulated genes, black dots: genes with no significant differences, green dots: down-regulated genes). Supplementary Figure 3. Differential expression analysis of ERSGs in the GSE4290 dataset. (A) Heap map of the differential expression of ERSGs in the GSE4290 set. (B) Volcano plot of DE-ERSGs in the GSE4290 dataset. (red dots: up-regulated genes, black dots: genes with no significant differences, green dots: down-regulated genes). Supplementary Figure 4. LASSO analysis based on the training set. (A) The minimum standard. (B) Coefficients. Supplementary Figure 5. Validation of differential expression of 8 genes by GEPIA database's data. Supplementary Figure 6. The testing results of model's performance based on GSE4412, GSE43378, and CGGA datasets. (A–C) The changes in sample's survival state with increasing risk score. (D–F) ROC curves reflecting model's prognostic predictive performance. (G–I) Kaplan–Meier survival curves reflecting model's prognostic discrimination performance. (J–L) The results of univariate Cox regression analysis to determine influencing factors of prognosis. (M–O) The results of multivariate Cox regression analysis to determine independent influencing factors of prognosis. Supplementary Figure 7. Calculate the relative importance of 8 PR-DE-ERSGs in glioma based on RF algorithm. (A–C) The error rates are influenced by the number of decision trees in three datasets, respectively. The *x*-axis represents the number of decision trees and the *y*-axis is the error rate. (D–F) The importance score of the 8 PR-DE-ERSGs in three datasets, respectively. The *x*-axis represents the importance index, and the *y*-axis represents the genes. (G–N) The ROC curves of 8 PR-DE-ERSGs from TCGA dataset. Supplementary Figure 8. Extending the model's clinical value. (A) Correlation analysis of risk scores with ICIs and m6A-related genes expression. (B–C) Differential expression analysis of ICIs and m6A-related genes in different risk-score subtypes, respectively. (D, F) Correlation analysis result between risk-score and ABCC1/ABCC3, respectively. (E, G) Differences in ABCC1/ABCC3 expression between different risk groups, respectively. Supplementary Table 1: the DE-FRGs used to construct the model and the corresponding coefficients. Supplementary Table 2. All primer sequences for qRT-PCR.
