# Supplementary material for: A Composite Bioinformatic Analysis to Explore Endoplasmic Reticulum Stress-Related Prognostic Marker and Potential Pathogenic Mechanisms in Glioma by Integrating Multiomics Data
Source: J Oncol. 2022 Oct 4;2022:9886044. doi: 10.1155/2022/9886044 (PMC9553508; doi:10.1155/2022/9886044)
Supplement: Supplementary Materials — Supplementary Figure 1. Differential expression analysis of ERSGs in the TCGA dataset. (A) Heap map of the differential expression of ERSGs in the TCGA dataset. (B) Volcano plot of DE-ERSGs in the TCGA dataset. (red dots: up-regulated genes, black dots: genes with no significant differences, green dots: down-regulated genes). Supplementary Figure 2. Differential expression analysis of ERSGs in the GSE108474 dataset. (A) Heap map of the differential expression of ERSGs in the GSE108474 set. (B) Volcano plot of DE-ERSGs in the GSE108474 set. (red dots: up-regulated genes, black dots: genes with no significant differences, green dots: down-regulated genes). Supplementary Figure 3. Differential expression analysis of ERSGs in the GSE4290 dataset. (A) Heap map of the differential expression of ERSGs in the GSE4290 set. (B) Volcano plot of DE-ERSGs in the GSE4290 dataset. (red dots: up-regulated genes, black dots: genes with no significant differences, green dots: down-regulated genes). Supplementary Figure 4. LASSO analysis based on the training set. (A) The minimum standard. (B) Coefficients. Supplementary Figure 5. Validation of differential expression of 8 genes by GEPIA database's data. Supplementary Figure 6. The testing results of model's performance based on GSE4412, GSE43378, and CGGA datasets. (A–C) The changes in sample's survival state with increasing risk score. (D–F) ROC curves reflecting model's prognostic predictive performance. (G–I) Kaplan–Meier survival curves reflecting model's prognostic discrimination performance. (J–L) The results of univariate Cox regression analysis to determine influencing factors of prognosis. (M–O) The results of multivariate Cox regression analysis to determine independent influencing factors of prognosis. Supplementary Figure 7. Calculate the relative importance of 8 PR-DE-ERSGs in glioma based on RF algorithm. (A–C) The error rates are influenced by the number of decision trees in three datasets, respectively. The x-axis rep [file 9886044.f1.docx]

# Supplementary figure legends


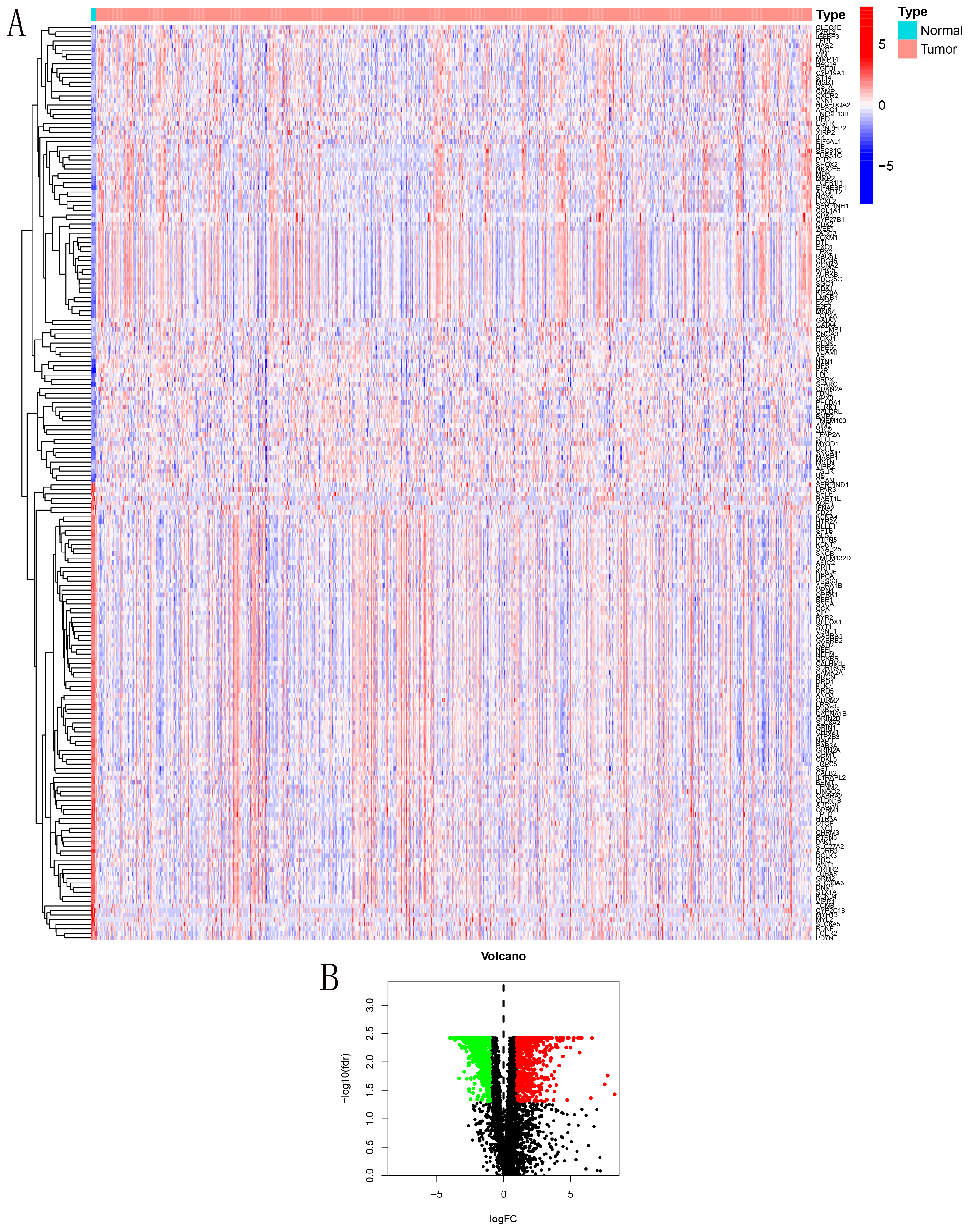


Supplementary Figure 1. Differential expression analysis of ERSGs in the TCGA dataset. (A) Heap map of the differential expression of ERSGs in the TCGA dataset. (B) Volcano plot of DE-ERSGs in the TCGA dataset. (red dots: up-regulated genes, black dots: genes with no significant differences, green dots: down-regulated genes).


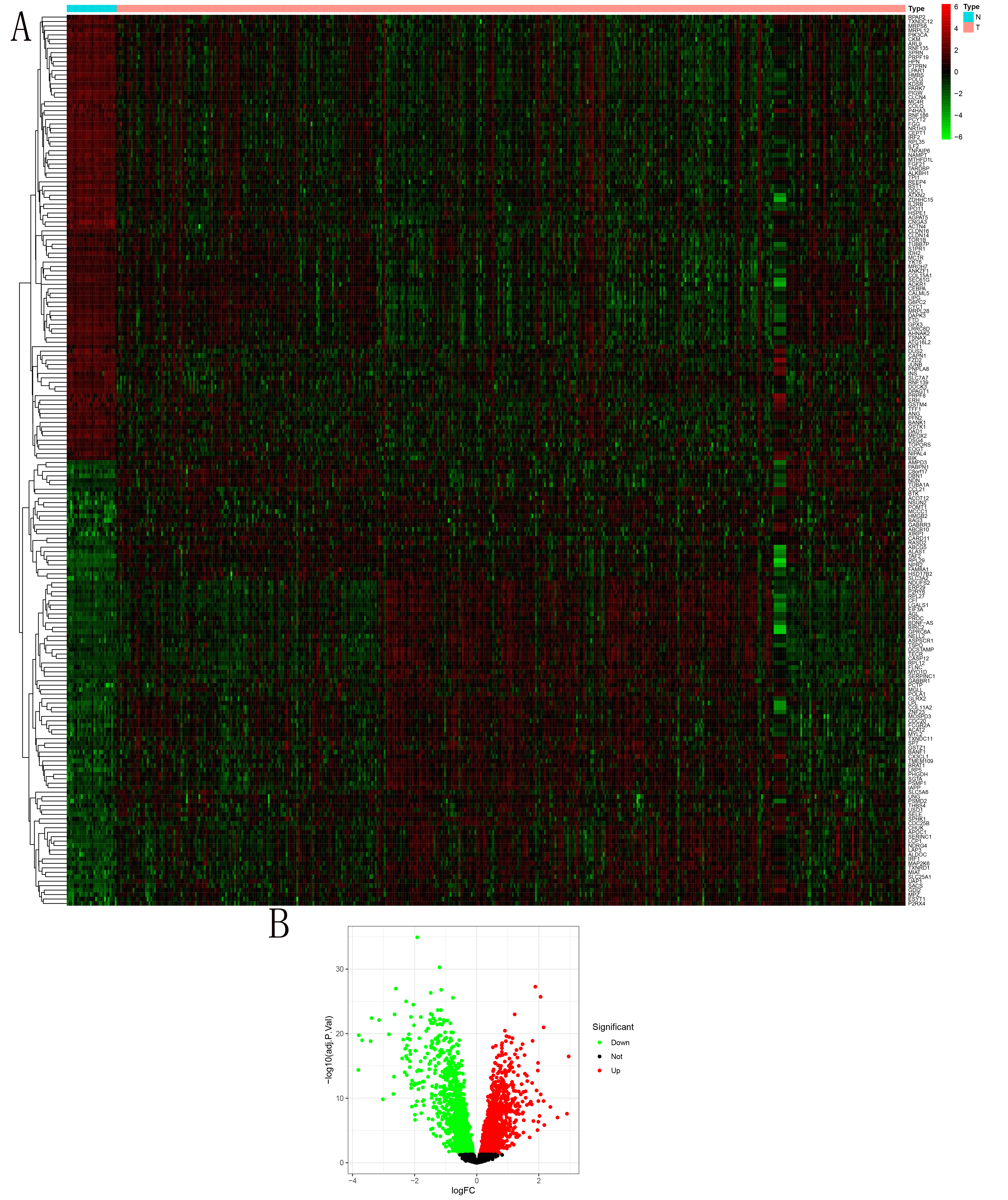


Supplementary Figure 2. Differential expression analysis of ERSGs in the GSE108474 dataset. (A) Heap map of the differential expression of ERSGs in the GSE108474 set. (B) Volcano plot of DE-ERSGs in the GSE108474 set. (red dots: up-regulated genes, black dots: genes with no significant differences, green dots: down-regulated genes).


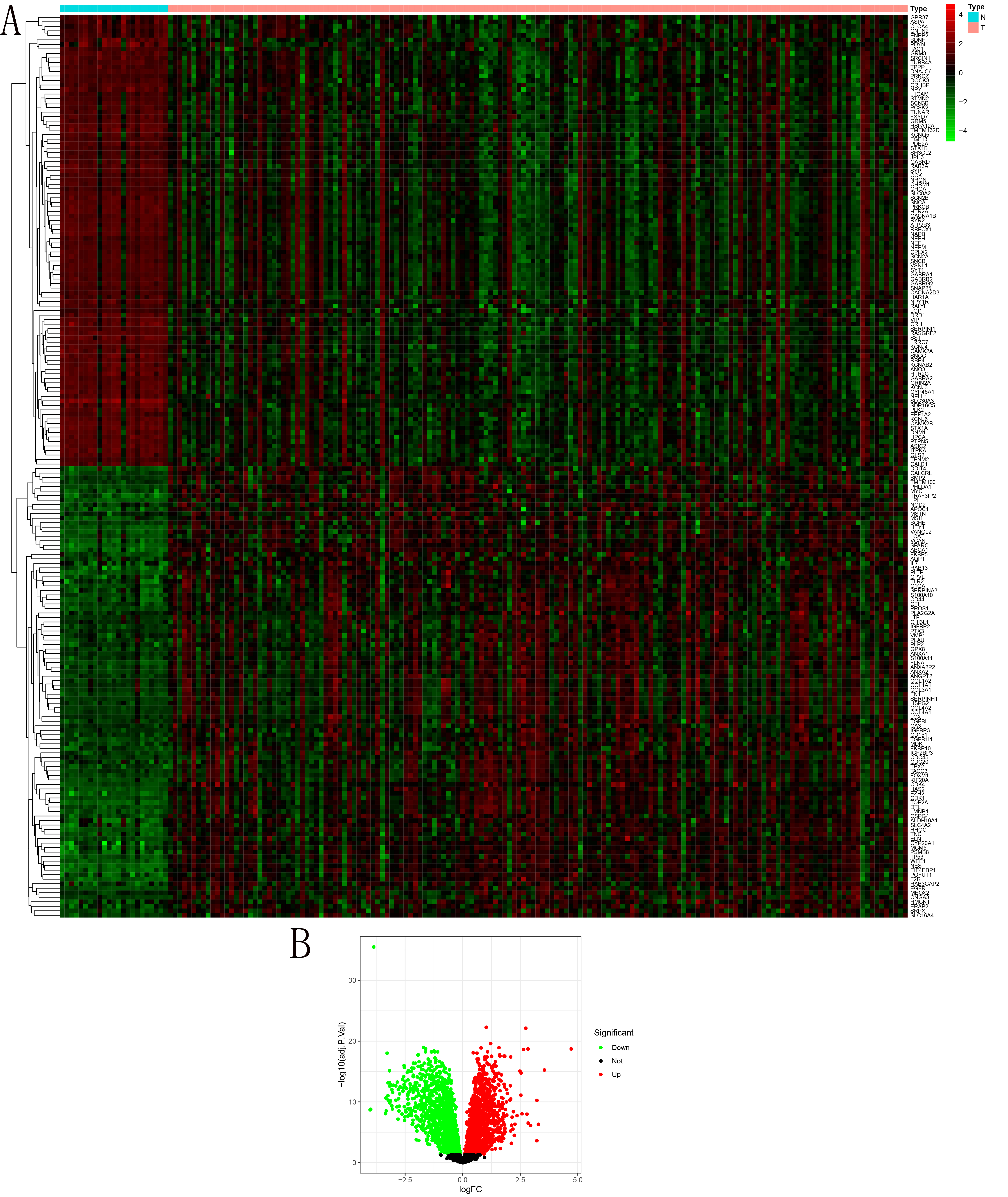


Supplementary Figure 3. Differential expression analysis of ERSGs in the GSE4290 dataset. (A) Heap map of the differential expression of ERSGs in the GSE4290 set. (B) Volcano plot of DE-ERSGs in the GSE4290. (red dots: up-regulated genes, black dots: genes with no significant differences, green dots: down-regulated genes).


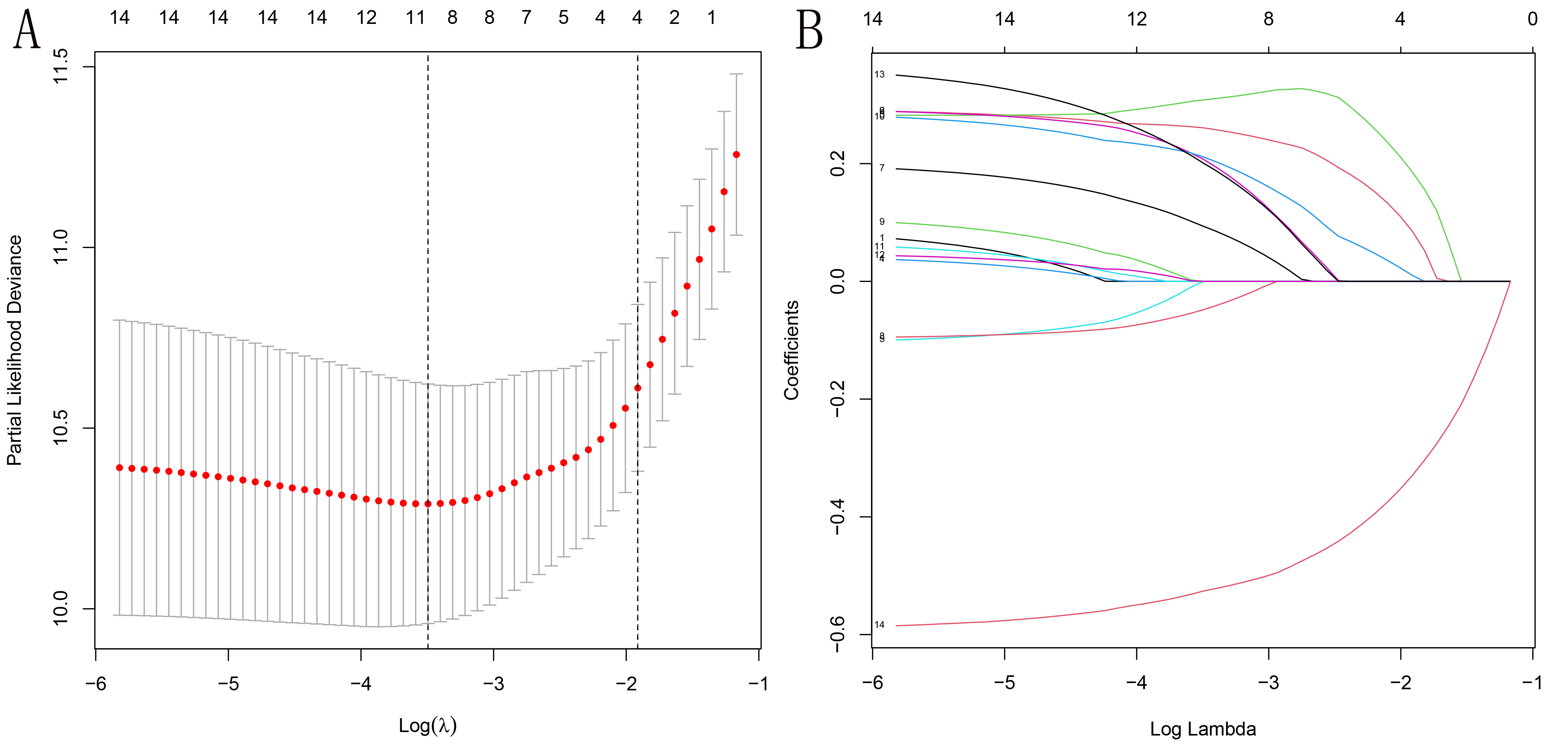


Supplementary Figure 4. LASSO analysis based on the training set. (A) The minimum standard. (B) Coefficients.


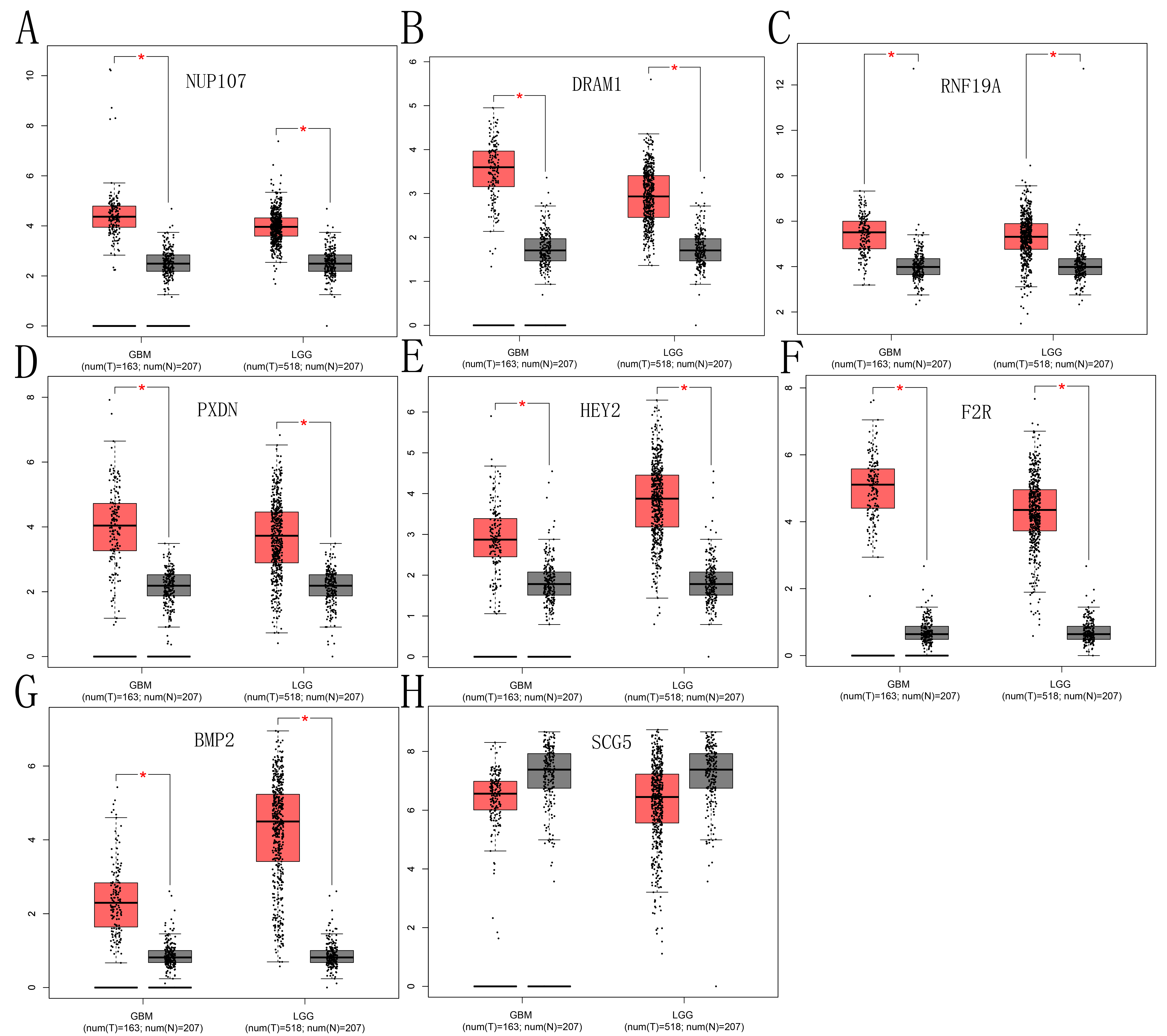


Supplementary Figure 5. Validation of differential expression of 8 genes by GEPIA database's data.


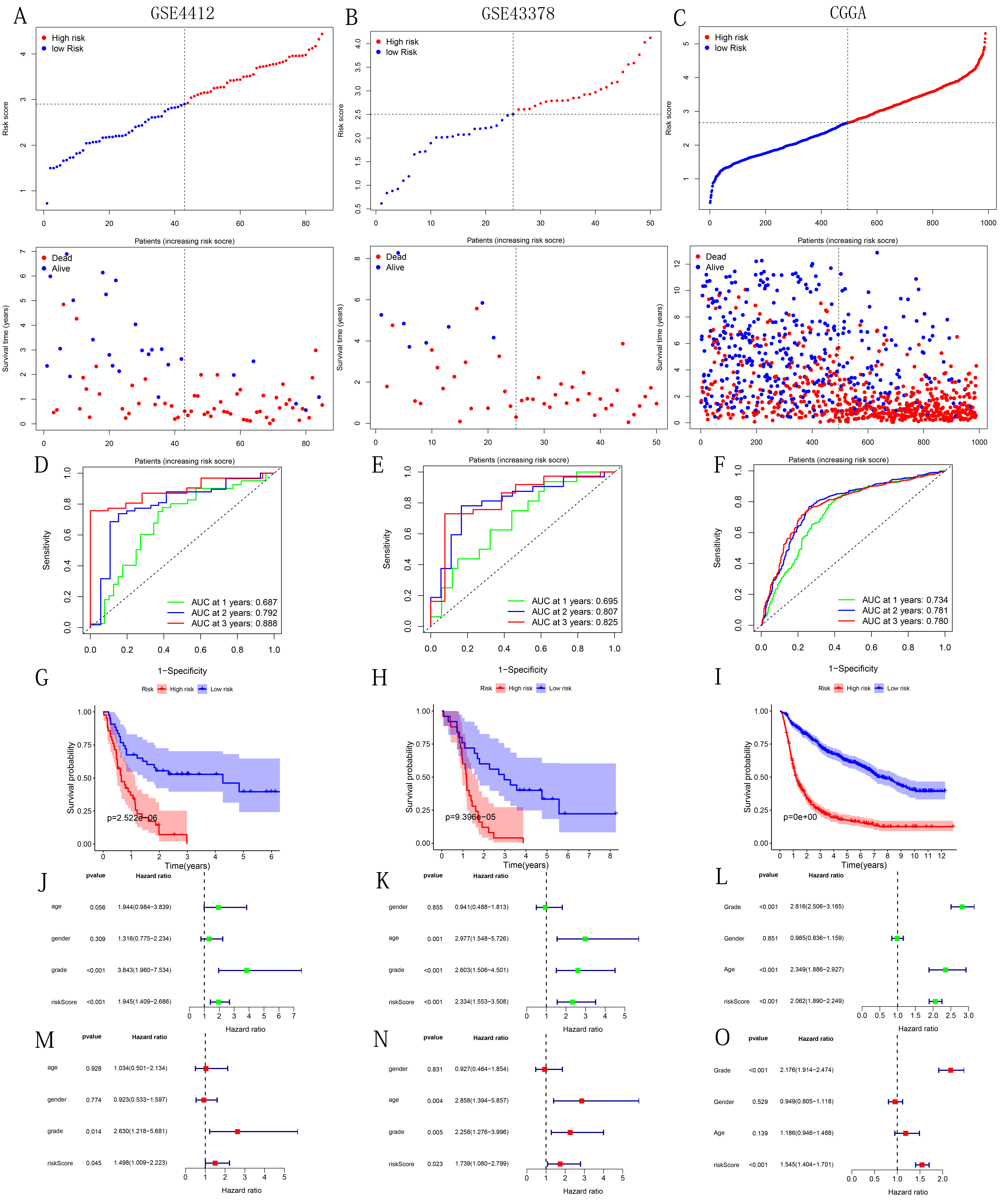


Supplementary Figure 6. The testing results of model's performance based on GSE4412, GSE43378 and CGGA datasets. (A-C) The changes in sample’s survival state with increasing risk score. (D-F) ROC curves reflecting model's prognostic predictive performance. (G-I) Kaplan-Meier survival curves reflecting model's prognostic discrimination performance. (J-L) The results of univariate Cox regression analysis to determine influencing factors of prognosis. (M-O) The results of multivariate Cox regression analysis to determine independent influencing factors of prognosis.


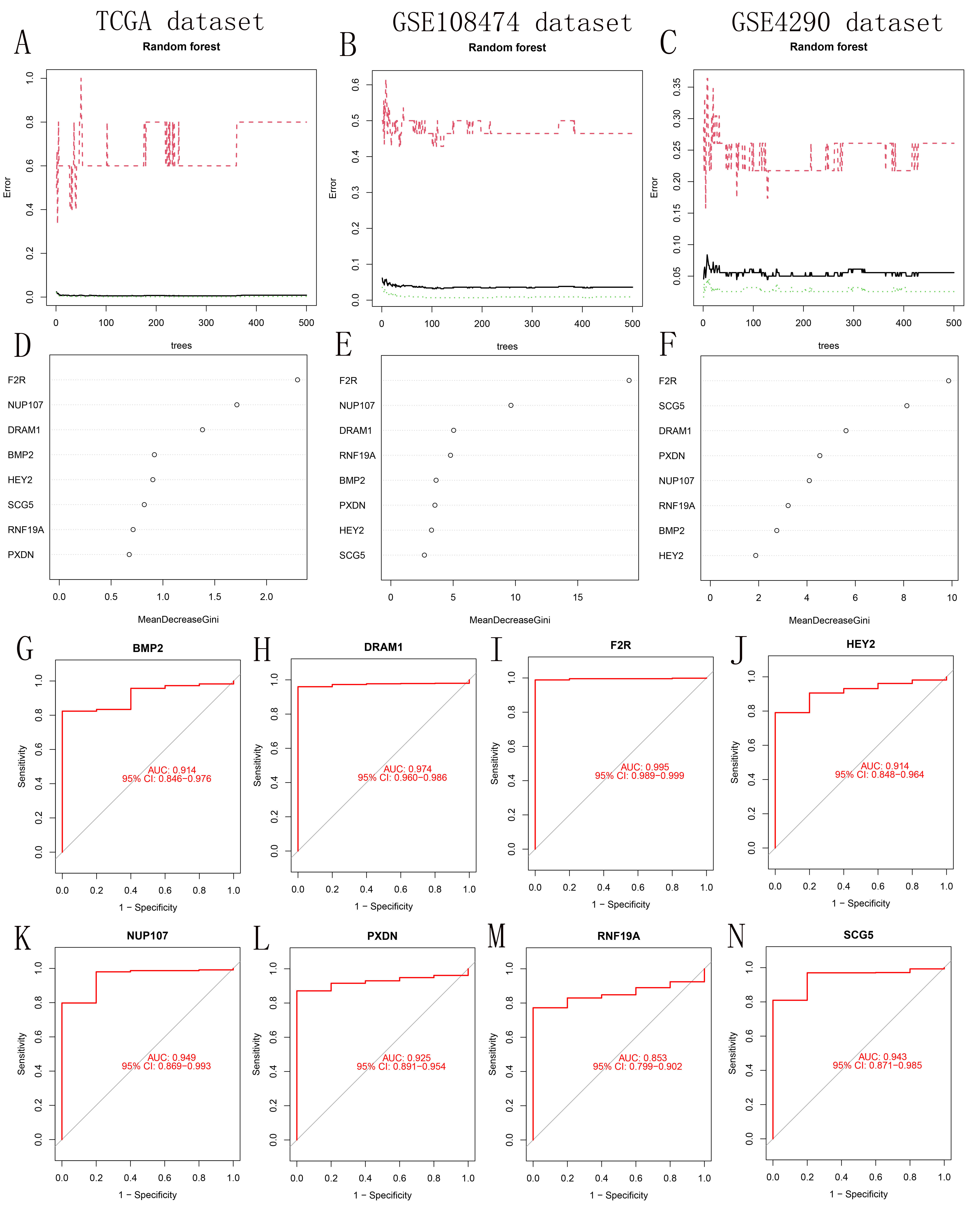


Supplementary Figure 7. Calculate the relative importance of 8 PR-DE-ERSGs in glioma based on RF algorithm. (A-C) The error rates are influenced by the number of decision trees in three datasets, respectively. The x-axis represents the number of decision trees and the y-axis is the error rate. (D-F) The importance score of the 8 PR-DE-ERSGs in three datasets, respectively. The x-axis represents the importance index, and the y-axis represents the genes. (G-N) The ROC curves of 8 PR-DE-ERSGs from TCGA dataset.


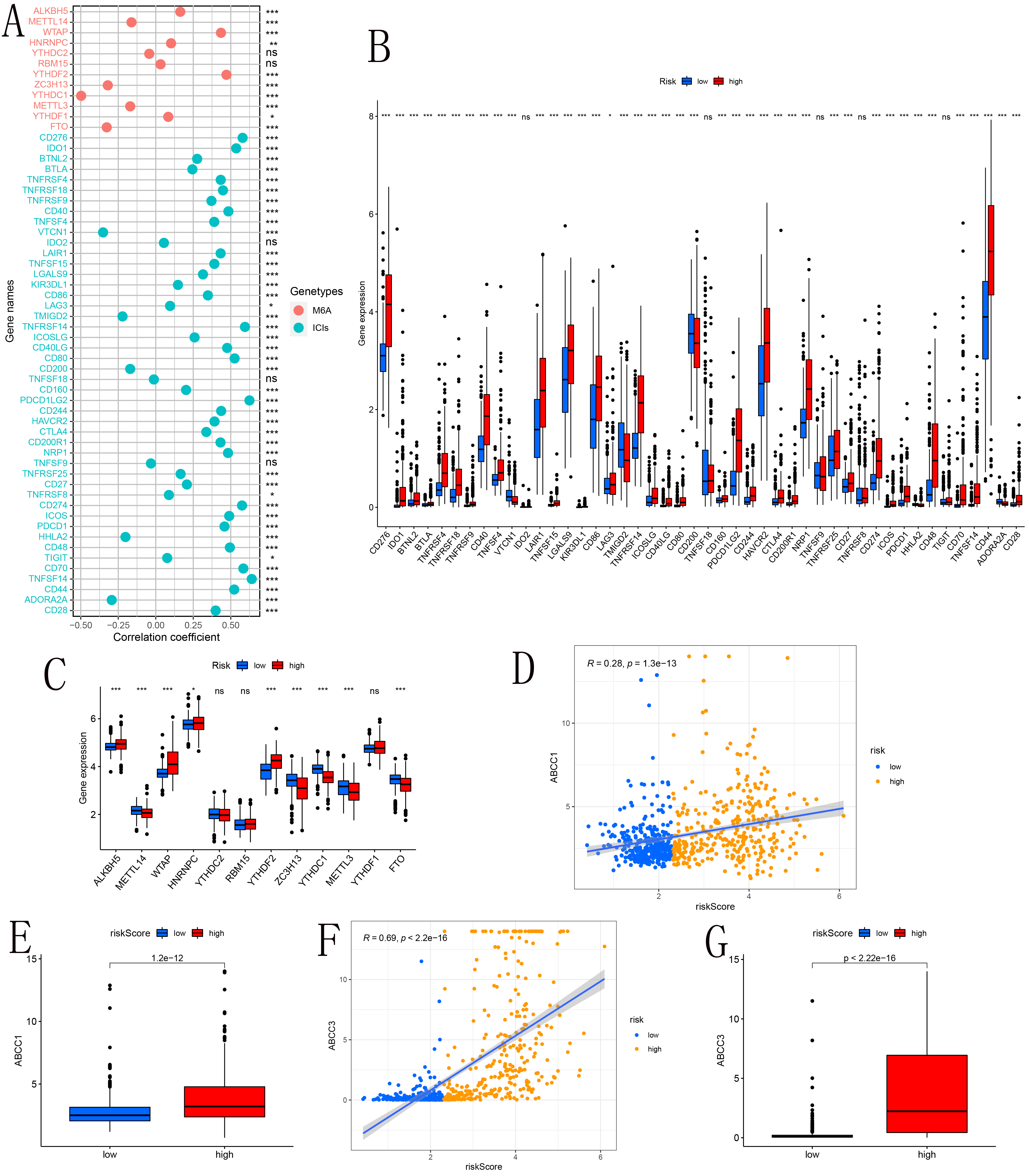


Supplementary Figure 8. Extending the model’s clinical value. (A) Correlation analysis of risk scores with ICIs and m6A related genes expression. (B-C) Differential expression analysis of ICIs and m6A related genes in different risk-score subtypes, respectively. (D, F) Correlation analysis results between risk-score and ABCC1/ABCC3, respectively. (E, G) Differences in ABCC1/ABCC3 expression between different risk groups, respectively.

# Supplementary Table

Supplementary Table 1. Eight PR-DE-ERSGs and corresponding coefficients by lasso regression.

| **Gene** | **Coefficient** |
| --- | --- |
| **NUP107** | 0.260812 |
| **DRAM1** | 0.30816 |
| **RNF19A** | 0.207429 |
| **PXDN** | 0.093586 |
| **HEY2** | -0.04814 |
| **F2R** | 0.211369 |
| **SCG5** | 0.200905 |
| **BMP2** | -0.52605 |

Supplementary Table 2. All primer sequences for qRT-PCR.

| **Gene** | Forward Primer | Reverse Primer |
| --- | --- | --- |
| **β-Actin** | TGGCACCCAGCACAATGAA | CTAAGTCATAGTCCGCCTAGAAGCA |
| **NUP107** | CACGGACTGCACGGAAACA | GAGTTCGAGGGATAACCTGGT |
| **DRAM1** | AGTGCTTGGATTGGTGGGATG | GATGGACTGTAGGAGCGTGTA |
| **SCG5** | ATGCTATCTGGCCTACTGTTTTG | GGCCCACAAGATTCATGGC |
| **BMP2** | ACCCGCTGTCTTCTAGCGT | TTTCAGGCCGAACATGCTGAG |
